# Adenovirus-mediated expression of orphan nuclear receptor NR4A2 targeting hepatic stellate cell attenuates liver fibrosis in rats

**DOI:** 10.1038/srep33593

**Published:** 2016-09-20

**Authors:** Pengguo Chen, Jie Li, Yan Huo, Jin Lu, Lili Wan, Quanjun Yang, Jinlu Huang, Run Gan, Cheng Guo

**Affiliations:** 1 Department of Pharmacy, Shanghai Jiao Tong University Affiliated Sixth People’s Hospital, 600 Yishan Road, Shanghai, China; 2 Shanghai Jiao Tong University School of Medicine, Shanghai, China; 3 Department of Gastroenterology, Jiangxi Provincial People’s Hospital, 92 Aiguo Road, Nanchang, Jiangxi, China

## Abstract

Liver fibrosis is a wound-healing response characterized with the accumulation of extracellular matrix (ECM). And hepatic stellate cells (HSCs) are the principal cell source of ECM. NR4A2 (Nurr1) is a member of orphan nuclear receptor NR4A family and acts as transcription factor. It participates in regulating cell differentiation, proliferation and apoptosis. We previously demonstrated that NR4A2 expression in fibrotic liver reduced significantly compared with normal liver and NR4A2 knockout in HSCs promoted ECM production. In the present study we explored the role of NR4A2 on liver fibrosis. Studies in cultured HSCs demonstrated that NR4A2 over-expression suppressed the activation of HSCs, such as ECM production and invasion ability. Moreover cell cycle was arrested, cell apoptosis was promoted and cell signaling pathway was influenced. Adenovirus-mediated delivery of NR4A2 in rats ameliorated significantly dimethylnitrosamine (DMN) induced liver fibrosis. The *In vivo* experiments produced results consistent with *in vitro* experiments. Taken together these results demonstrate NR4A2 enhancement attenuates liver fibrosis via suppressing the activation of HSCs and NR4A2 may be an ideal target for anti-fibrotic therapy.

Liver fibrosis is a wound-healing response characterized with the accumulation of extracellular matrix (ECM). An increase in the amount of the ECM is the typical feature of all forms of fibrosis. During liver fibrogenesis there is significant increase in the content of collagens, particularly of fibril-forming types I and III^1^. The reiterated liver tissue damage due to infective (mostly hepatitis B and C viruses), toxic induced, metabolic and autoimmune can last for several decades and leads to cirrhosis, the end consequence of hepatic fibrosis, which has high mortality^2^.

Epithelial mesenchymal transition (EMT) has been closely related to liver fibrogenesis through which epithelial cells contribute to the replacement of dead or damaged hepatic cells^3^,^4^,^5^. The ECM is mostly produced by myofibroblasts. HSCs (hepatic stellate cells) are the key myofibroblast population in hepatic fibrosis. It was reported that 94% to 96% of myofibroblasts were derived from HSCs in liver fibrotic models and the contribution of HSCs to the hepatic myofibroblast pool in liver fibrosis ranged from 82% to 89% 6.

HSCs reside in the space of Disse which constitute 5–15% of all hepatic cells and represent a vital fibrogenic cell population in liver. HSCs store retinyl esters in intracytoplasmic lipid droplets and exhibit features of vascular pericytes regulating sinusoidal blood flow and produce significantly more ECM than parenchymal cells^7^,^8^,^9^.

Both in animal models and human beings, it has been proved that liver fibrosis is reversible with treatment not limited to cessation of the causative agent^10^,^11^,^12^,^13^. Targeting both specific functions of myofibroblast (collagen production) and myofibroblast themselves might prove therapeutically effective. HSCs are not only a key fibrogenic cell population in the liver but also amenable to cell-specific delivery approaches which makes them an attractive candidates for direct anti-fibrotic therapy^14^.

NR4A2 (also called Nurr1) is a member of orphan nuclear receptor NR4A family and acts as transcription factor. The NR4A subfamily members are widely distributed in cells regulating differentiation, proliferation and apoptosis and involved in multiple diseases, for example vascular sclerosis, cancer and metabolic syndrome. Our previous study demonstrated NR4A2 knockdown could promote HSCs proliferation^15^. In this study we explored the effect of AdNR4A2, adenovirus carrying NR4A2 gene, on activated HSCs or liver fibrosis and clarified its mechanism. Our data showed that NR4A2 gene over-expression by adenovirus-mediated significantly suppressed the activated HSCs and attenuated dimethylnitrosamine (DMN) reduced hepatic fibrosis. These results suggest that NR4A2 could be a promising target gene for anti-fibrotic therapy and exploiting agents that increases NR4A2 expression would be very meaningful.

## Results

### AdNR4A2 decreases the expression of ECM gene in HSCs

In liver fibrosis, activated HSCs produced most ECM and the level of α smooth muscle actin (α-SMA). a typical marker for liver fibrosis elevates significantly.

**Figure 1:**
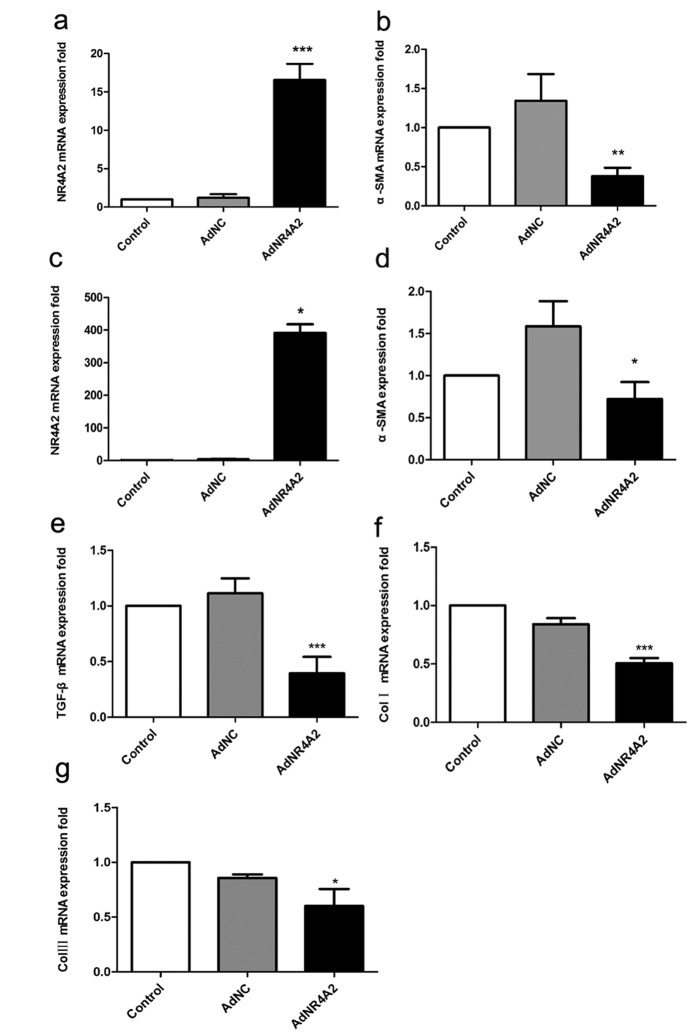
AdNR4A2 treatment causes increased NR4A2 expression and decreased α-SMA expression in hepatic stellate cells. HSC-T6 cells or rat primary hepatic stellate cells were treated with AdNR4A2 and AdNC respectively at an MOI of 40 for 72 hours. (**a**) The NR4A2 mRNA level in HSC-T6 cells. (**b**)The α-SMA mRNA level in HSC-T6 cells. (**c**)The NR4A2 mRNA level in rat primary hepatic stellate cells. (**d**) The α-SMA mRNA level in rat primary hepatic stellate cells. (**e**) The TGF-β mRNA level in HSC-T6 cells. (**f**) The Col I mRNA level in HSC-T6 cells. (**g**) The Col III mRNA level in HSC-T6 cells. The values are expressed as the mean ± SD (n = 3). *p < 0.05, **p < 0.01 and ***p < 0.001 vs. AdNC group.

To investigate the effect of NR4A2 on ECM production AdNR4A2 and its negative control AdNC were generated. We verified the MOI (Multiplicity of Infection) of AdNR4A2 and AdNC in HSCs and the preferred MOI was 40. RT-PCR analysis showed the NR4A2 mRNA expression in HSCs infected with AdNR4A2 was 100 times higher than in cells infected with AdNC (Fig. 1a). Meantime theα-SMA level decreased significantly by 70% (Fig. 1b). The results demonstrated that ECM production was restrained after NR4A2 enhancement in HSC-T6 cells.

Furthermore we investigated the influence of AdNR4A2 on primary hepatic stellate cells. Primary hepatic stellate cells could activate spontaneously after isolation that resembles the activation process *in vivo*^16^. We isolated primary hepatic stellate cells from SD-rats and on the third day AdNR4A2 or AdNC infected the primary hepatic stellate cells respectively. RT-PCR results showed that NR4A2 mRNA level increased by 300-fold and α-SMA level reduced by 50% in AdNR4A2 treated cells than in AdNC treated cells (Fig. 1c,d). These data implied that NR4A2 may inhibit ECM production *in vivo*.

TGF-β plays a central role in activating HSCs and induces the transcription of type I and III collagen which are important components of ECM^17^. RT-PCR analysis showed the TGF-β mRNA expression reduced by more than 60% in AdNR4A2 treated cells than in AdNC treated cells (Fig. 1e). Meanwhile the Col I and Col III level decreased by 40% and 20% respectively after NR4A2 enhancement in HSCs (Fig. 1f,g).

### AdNR4A2 arrests cell cycle and promotes HSCs apoptosis

**Figure 2:**
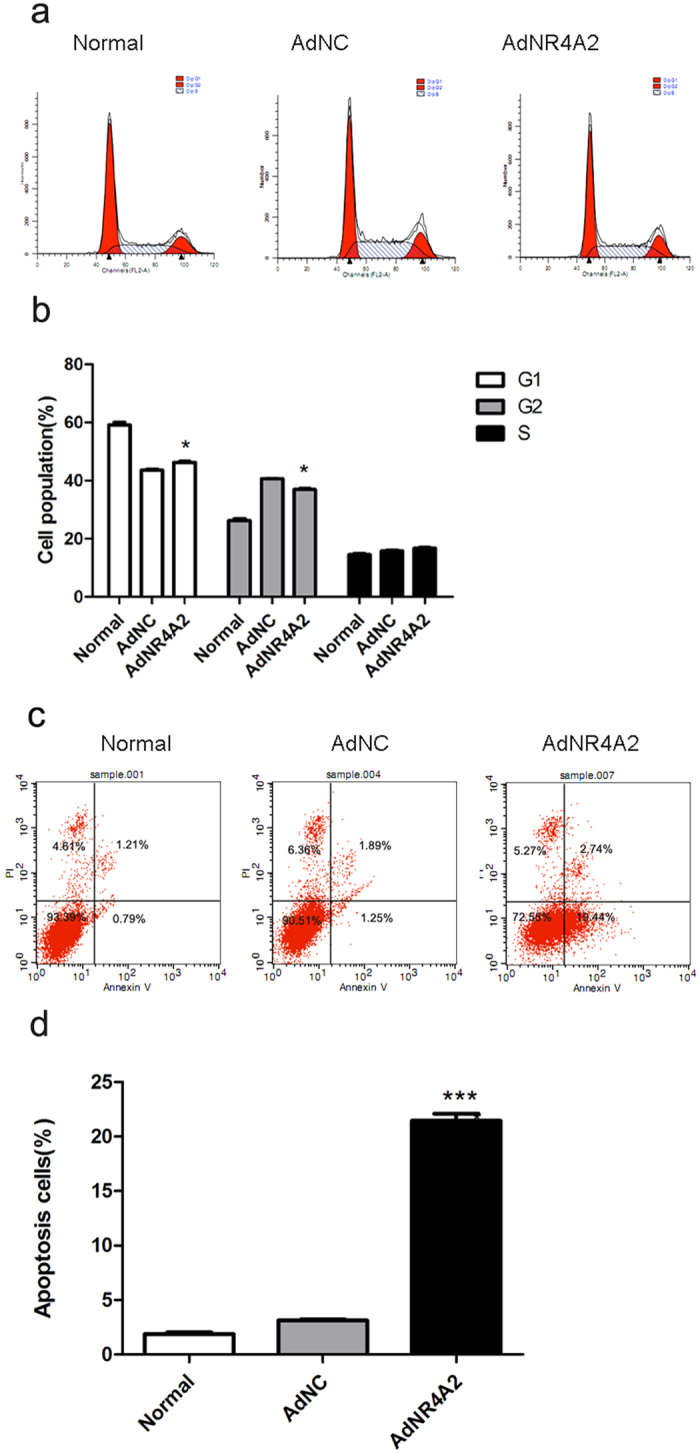
AdNR4A2 treatment causes cell cycle arrest and increased cell apoptosis rate. **(a,b)** HSC-T6 cells treated with AdNR4A2 and AdNC respectively at an MOI of 40 for 72 hours were stained with propidium iodide and analyzed by FACS to determine cell cycle. (**a**) Representative flow cytometry graph for control, AdNC and AdNR4A2 group. (**b**) Summary of cell cycle. (**c**,**d**) HSC-T6 cells treated with AdNR4A2 and AdNC respectively at an MOI of 40 for 72 hours were stained with propidium iodide and subjected to FACS analysis for determination of apoptotic cells. (**c**) Representative apoptosis graph for control, AdNC and AdNR4A2 group. (**d**) Summary of cell apoptosis. The values are expressed as the mean ± SD (n = 3). *p < 0.05 and ***p < 0.001 vs. AdNC group.

During fibrosis reversion stage activated HSCs revert to quiet state or even apoptosis. To explore whether AdNR4A2 promote cell apoptosis or prohibit cell proliferation, AdNR4A2 or AdNC infected HSCs respectively for 48 hour. FACS analysis of propidium iodide-stained cells revealed that AdNR4A2 treatment caused significant cellular accumulation in G1 phase and reduced percentage in S phase compared with AdNC treatment (Fig. 2a,b and Supplementary Fig. S1a,b,c).

Meanwhile we investigated the influence of AdNR4A2 on cell apoptosis. FACS for cell apoptosis assay showed that cell apoptosis rate increased significantly from about 2–3% to 22% in AdNR4A2 infected cells than in AdNC infected cells (Fig. 2c,d and Supplementary Fig. S1d,e,f). Above results confirmed that AdNR4A2 promoted markedly cell apoptosis apart from arresting cell cycle in HSCs.

### AdNR4A2 inhibits cell migration

In liver fibrogenesis HSCs also contribute to angiogenesis regeneration. Activated HSCs migrate along with endothelial cells to establish vascular connections which lead to creating new sinusoidal branches^18^.

**Figure 3:**
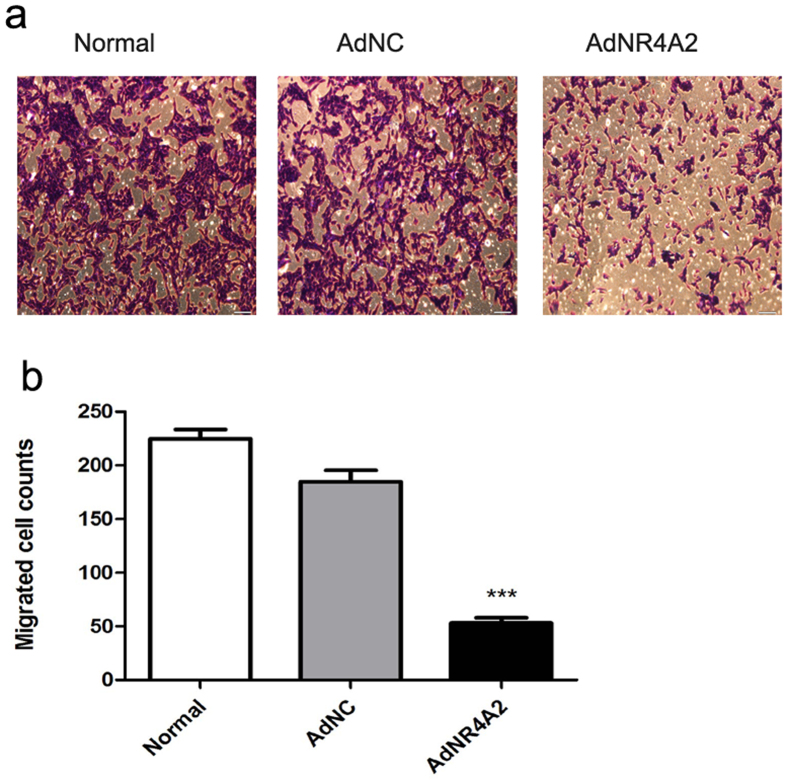
AdNR4A2 treatment inhibits the migration and invasion ability in hepatic stellate cells. HSC-T6 cells treated with AdNR4A2 and AdNC respectively at an MOI of 40 for 72 hours were subjected to transwell migration assay. (**a**) Representative Transwell graph for control, AdNC and AdNR4A2 group. (**b**)Analysis for the number of migrated cells. The values are expressed as the mean ± SD (n = 3). ***p < 0.001 vs. AdNC group. Scale bar 200 um.

In order to assess whether AdNR4A2 inhibit HSCs from migrating transwell migration assays were employed. Transwell results showed that cell migration ability was weakened significantly after AdNR4A2 treatment (Fig. 3a,b). It implied that AdNR4A2 could suppress the migration of activated HSCs.

### AdNR4A2 increases the phosphorylation of ERK1/2 and P38 in HSCs

During the activation of HSCs multiple signaling pathways play important role. And among them MAPK pathway is vital for the activation of HSCs^19^. MAPK family includes extracellular signal-regulated kinase (ERK), c-Jun N-terminal kinase (JNK) and P38.

**Figure 4:**
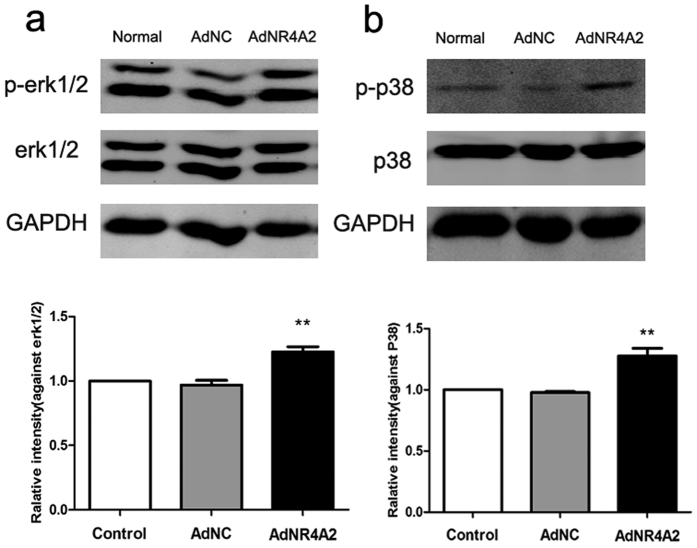
AdNR4A2 treatment increases the phosphorylation of ERK1/2 and P38 in hepatic stellate cells. Western blots were performed in HSC-T6 cells treated with AdNR4A2 and AdNC respectively at an MOI of 40 for 72 hours. (**a**) Western blot analysis on reducing blots and quantitative measurement of phosphorylation of ERK1/2. (**b**) Western blot analysis on reducing blots and quantitative measurement of phosphorylation of P38.The values are expressed as the mean ± SD (n = 3). **p < 0.01 vs. AdNC group.

We explored the effect of AdNR4A2 on phosphorylation of MAPK. AdNR4A2 or AdNC infected HSC-T6 cells respectively for 72 hour. The Western blot results showed increased phosphorylation of ERK and P38 after AdNR4A2 treatment (Fig. 4a,b). The data suggested that AdNR4A2 may activate MAPK signaling pathway in HSCs to suppress the activated HSCs.

### AdNR4A2 increases the export of nucleus of NR4A2

Orphan nuclear receptor NR4A2 mostly locates in nucleus. The translocation from nucleus to cytoplasm, such as mitochondria, triggers release of cytochrome C which results in the cell apoptosis^20^. To verify the correlation between AdNR4A2 promoted cell apoptosis and the nucleus export of NR4A2 in HSCs cell immunofluorescence assay was performed.

**Figure 5:**
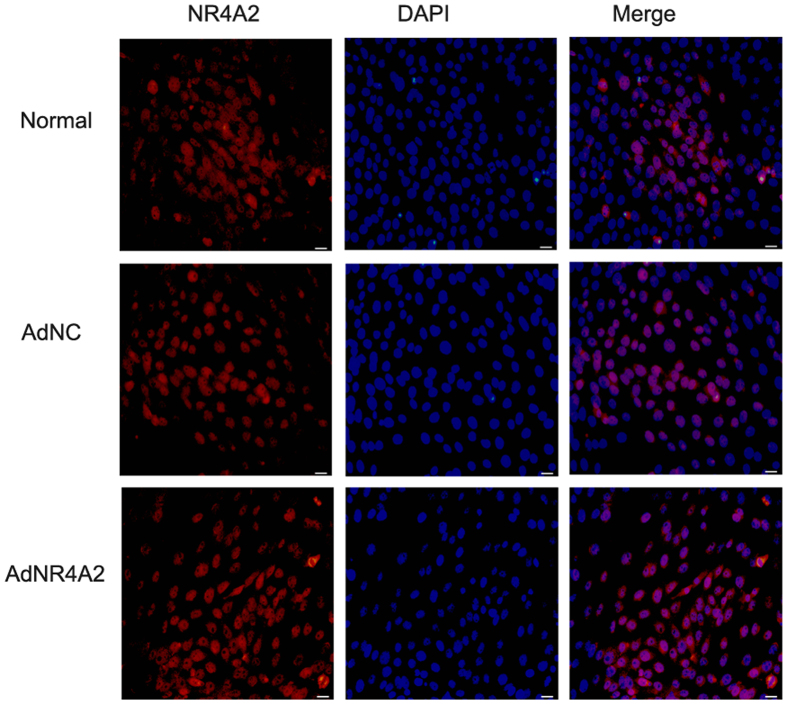
AdNR4A2 treatment leads the cytoplastic translocation of NR4A2 in hepatic stellate cells. HSC-T6 cells treated with AdNR4A2 and AdNC respectively at an MOI of 40 for 72 hours were subjected to immunofuorescent costaining for NR4A2. Counterstaining was performed using DAPI for detection of nuclei. Cell fluorescence results showed cytoplastic translocation of NR4A2 in HSC-T6 cells. Representative photomicrographs are shown at ×20 magnification. scale bar 100 μm.

Our results indicated the rate of NR4A2 nuclear translocation in AdNR4A2 treated cells was higher than in AdNC treated cells (Fig. 5).

### AdNR4A2 attenuates DMN reduced liver fibrosis

**Figure 6:**
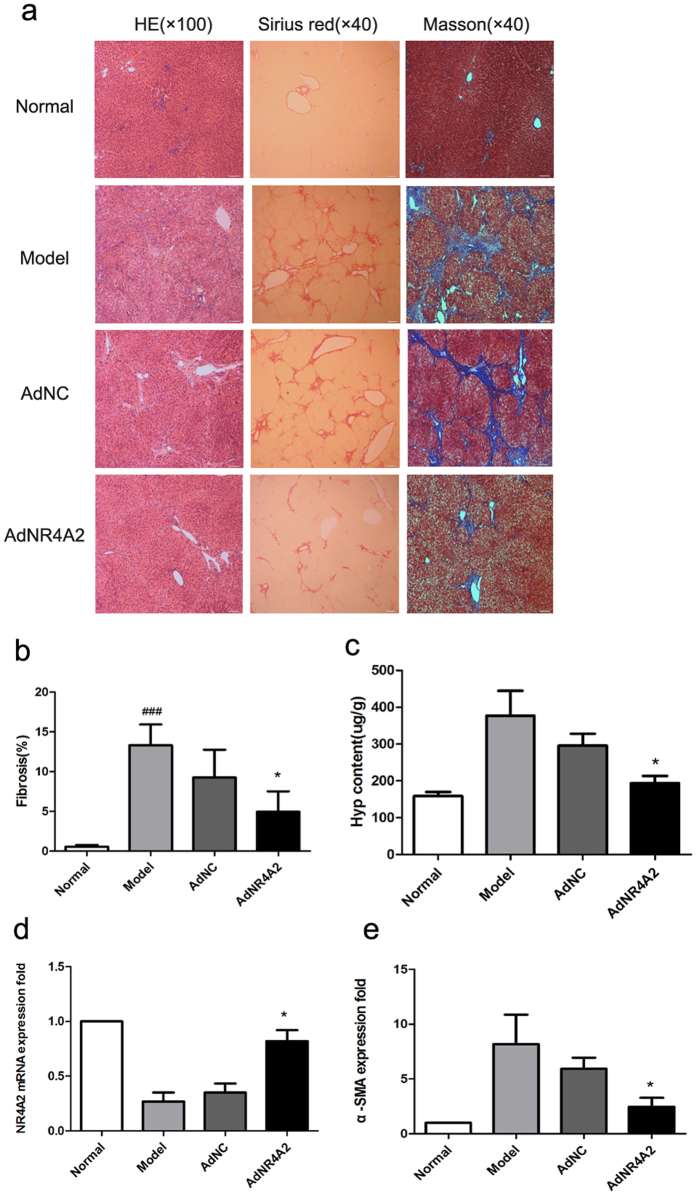
AdNR4A2 treatment alleviates dimethyl nitrosamine-induced liver fibrosis. Rats harboring dimethyl nitrosamine-induced hepatic fibrosis were treated by infusion of AdNR4A2, AdNC and medium respectively and sacrificed. The normal healthy rats were also sacrificed meantime. Paraffin-embedded liver sections were visualized by H/E staining, Sirius Red and Masson’s trichrome staining and representative fields of view were photographed at ×4 or ×10 magnification. Stage 3 fibrosis is exemplarily shown in the medium-treated or AdNC-treated panel, as evidenced by fibrous bridges connecting portal tracts and central veins with formation of multiple nodules whereas there was no fibrous tissue in normal healthy rat livers. The AdNR4A2-treated bottom panel is representative of stage 2 fibrosis, in which connective tissue links neighboring portal tracts while the overall architecture is preserved (**a**). (**b**) The Masson’s trichrome staining results in normal rats, AdNR4A2 group, AdNC group and model group were subjected to fibrosis score analysis by computerized image morphometric analysis. (**c**) Collagen content (per g of liver) in normal rats, AdNR4A2 group, AdNC group and model group were measured by hydroxyproline biochemical determination. (**d,e**) mRNAs extracted from liver in normal group, AdNR4A2 group, AdNC group and model group were reverse-transcribed and used as template for quantitative real-time PCR (qPCR) to determine the relative NR4A2 and α-SMA expression level. AdNR4A2, AdNC and medium-treated samples and normal healthy sample were presented in terms of their relative expression level with respect to the normal control. (**d**) NR4A2 level in liver tissue. (**e**) α-SMA level in liver tissue. The values are expressed as the mean ± SD (n = 6). *p < 0.05 vs. AdNC group. ^###^p < 0.001 vs. model group

Further, we studied the effect of AdNR4A2 against DMN-induced liver fibrosis in rats. The rats were sacrificed and hepatic fibrosis was evaluated by hematoxylin and eosin, Sirius red and Masson’s trichrome staining. The rats in AdNC (AdNC and DMN treated together) group and model (DMN treated only) group exhibited evident and uniform liver fibrosis after 4 weeks of repeated DMN injection. And most rats in the two groups showed stage 3 or 4 liver fibrosis. The severity of the liver fibrosis was markedly milder in the AdNR4A2 (AdNR4A2 and DMN treated together) group than that in AdNC group or model groups with most indicating stage 2 liver fibrosis (Fig. 6a and Supplementary Fig. S2).

Fibrosis score for the Masson’s trichrome staining suggested that fibrosis area increased significantly in AdNC group or model group after hepatic fibrosis was induced. At the same time the fibrosis area decreased evidently after AdNR4A2 treatment (Fig. 6b).

Hydroxyproline content, a commonly accepted indicator of fibrous tissue, was determined in the liver tissue. Accordance with the histological grading the liver hydroxyproline content of the AdR4A2 group was significantly lower than that of AdNC group or model group, and there was no significant difference between AdNC group and model group (Fig. 6c).

RT-PCR analysis for mRNA of rat liver tissue indicated that α-SMA level increased obviously and NR4A2 level reduced sharply in model group or AdNC group than in normal group. The NR4A2 level elevated andα-SMA level reduced significantly in AdNR4A2 group compared with AdNC group or model group (Fig. 6d,e).

**Figure 7:**
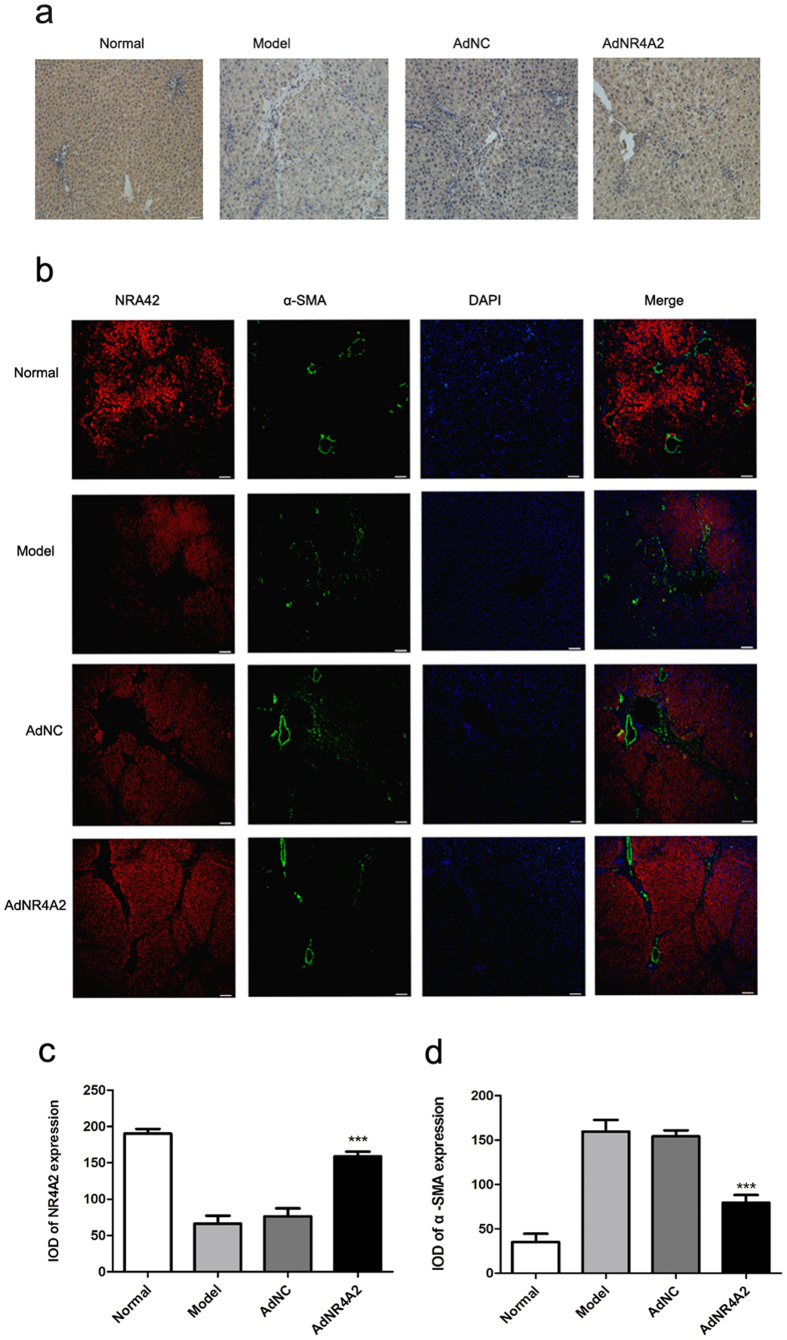
AdNR4A2 treatment increases NR4A2 level and decreases α-SMA level in dimethyl nitrosamine-induced fibrotic liver tissue. Rats harboring dimethyl nitrosamine-induced hepatic fibrosis were treated by infusion of AdNR4A2, AdNC and medium respectively and sacrificed. The normal healthy rats were also sacrificed meantime. Paraffin-embedded liver sections were made. (**a**) Immunohistochemical staining was performed with an antibody specific for NR4A2 and representative sections were imaged at ×20 magnification. scale bar 100 μm. (**b**) Immunofuorescent costaining for NR4A2 and α-SMA. scale bar 200 μm. (**c,d**) The bar graphs showed the statistical results for NR4A2 and α-SMA expression in immunofluorescent images. The values are expressed as the mean ± SD (n = 6). ***p < 0.001 vs. AdNC group.

We detected the NR4A2 and α-SMA protein expression in liver tissue. Immunohistochemisty staining showed a sharp reduced staining of NR4A2 around portal tracts and in fibrotic septa after DMN injection. And the staining of NR4A2 increased significantly in AdNR4A2 group compared with AdNC group or model group (Fig. 7a). Immunofluorescence staining results revealed decreased staining of NR4A2 around portal tract and fibrotic septa after DMN injection, which was accompanied by increased α-SMA expression, implying that AdNR4A2 is involved in the inactivation of hepatic stellate cells. In consequence increased staining of NR4A2 accompanied by decreased expression of α-SMA was found in AdNR4A2 group than in AdNC group or model group (Fig. 7b and Supplementary Fig. S3). The staining of NR4A2 by double –label immunofluorescence in AdNR4A2 group was more intense than that in model group or AdNC group. There was no significant difference between model group and AdNC group (Fig. 7c). Meanwhile, the immunofluorescence of α-SMA in AdNR4A2 group exhibited lower density compared with model group or AdNC group (Fig. 7d).

**Figure 8:**
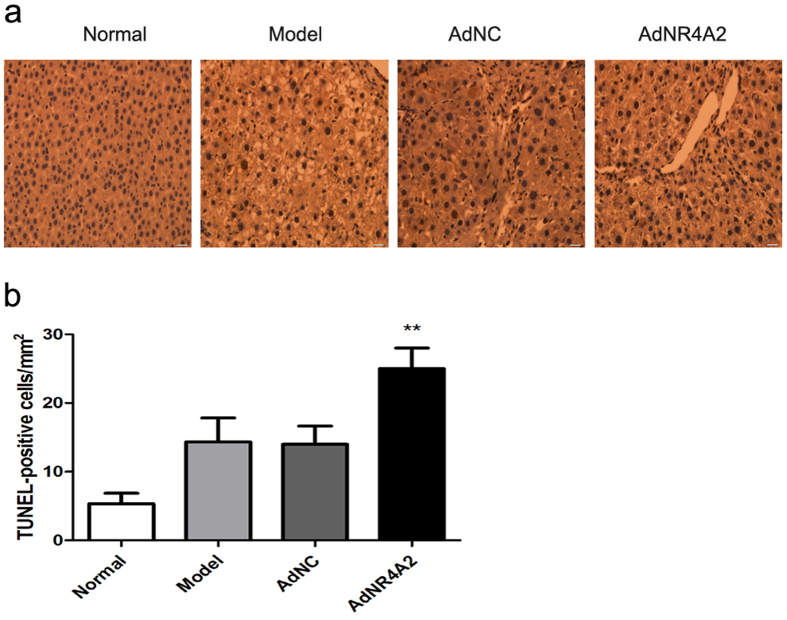
AdNR4A2 treatment promotes apoptosis in *vivo* in rats. Rats harboring dimethyl nitrosamine-induced hepatic fibrosis were treated by infusion of AdNR4A2, AdNC and medium respectively and sacrificed. The normal healthy rats were also sacrificed meantime. Paraffin-embedded liver sections were subjected to TUNEL assay. (**a**) Representative TUNEL graph for normal group, model group, AdNC group and AdNR4A2 group. (**b**) The number of apoptosis cells in liver tissue. The values are expressed as the mean ± SD (n = 6). **p < 0.01 vs. AdNC group. scale bar 100 μm.

To identify the effect of AdNR4A2 on apoptosis *in vivo*, we performed a staining for TUNEL in liver tissue. Of note, apoptotic cells were hardly observed in normal group. The number of apoptotic cells increased in model group or AdNC group after liver fibrosis was induced, but no variance occurred between them. And the number of apoptotic cells elevated significantly after AdNR4A2 treatment than in AdNC group or model group (Fig. 8a,b). These data implied that AdNR4A2 therapy may lead to the cell apoptosis *in vivo*.

## Discussion

Liver fibrosis is the result of sustained hepatic inflammation and characterized with large amount of ECM in which HSCs play an important role. Quiet HSCs can be activated by a variety of insults. Activated HSCs secrete cytokines such as TGF-β, IL-6, IL-8, COX2 and TLR4. Among them TGF-β is a central regulator of mesenchymal repair responses and orchestrates the release of extracellular matrix during tissue repair^17^. Both reverting activated HSCs to quiet state or apoptosis and inhibiting profibrogenic gene expression of HSCs are effective for anti-fibrotic therapy. Recently it was demonstrated that liver fibrosis is reversible in either human being or animal model^10^,^11^,^12^,^13^.

Accumulated evidence suggested nuclear receptors regulate key steps in activation of HSCs^21^. Peroxisome proliferator activated receptor-γ (PPAR-γ) inhibits profibrogenic genes expression in quiescent HSCs. Treatment with PPAR-γ agonists restrains HSCs from activation and suppresses collagen production^22^,^23^. Our previous study indicated that NR4A2 level decreased significantly in fibrotic liver or activated HSCs and knockout of NR4A2 led to the increase of α-SMA and collagen I 15. Our current study showed that NR4A2 gene over-expression resulted in the reduction of *α*-SMA in HSC-T6 cells. In addition, we obtained the same results in primary hepatic stellate cells. These cells could be spontaneously stimulated after isolation in which quiet HSCs transform into activated HSCs which resembles the activation process *in vivo*^16^.

TGF-β is a master regulator of mesenchymal responses in physiological and pathological conditions and induces the transcription of type I and III collagen in HSCs, promoting liver fibrosis^17^. Our previous study showed when HSCs were transiently stimulated by TGF-β the NR4A2 expression increased. Yet persistent stimulation led to reduced NR4A2 level^15^. We further demonstrated that NR4A2 over-expression resulted in decreased levels of TGF-β, Col I and Col III in HSCs. It implied that NR4A2 is a key checkpoint to control TGF-β signaling and chronically activated TGF-β signaling escapes the regulatory effects of NR4A2 by disrupting the NR4A2 feedback loop. Adenovirus mediated NR4A2 enhancement could re-activate an endogenous regulatory loop. When TGF-β expression was inhibited the levels of Col I and Col III reduced in the end. But the detailed mechanism needs to explore in future study.

NR4A2 is involved in regulating cell cycle and cell apoptosis^24^. In our study, AdNR4A2 treatment led to cell cycle arrest such as increased percentage of cells in G1 phase and reduced percentage of cells in G2 phase as well as significant increase of cell apoptosis rate. It implied that NR4A2 enhancement could revert activated HSCs to quiet state via arresting cell cycle and promote cell apoptosis directly. This is consistent with documents that NR4As induces cell death apart from as transcription factor regulating target gene expression^25^,^26^,^27^.

NR4A2 can relocate from nucleus to mitochondria and bind to Bcl-2 which triggers cytochrome C release and results in cell apoptosis^20^. We applied adenovirus carrying NR4A2 to infect HSCs and found that the rate of NR4A2 nuclear translocation increased significantly. To a certain degree our study consistent with previous reports, but the mechanism and details require further exploration.

During liver fibrosis activated HSCs can migrate vigorously which is a distinctive property of activated HSCs^28^. Our study showed that NR4A2 enhancement can weaken its migration and invasion ability. It illustrated that NR4A2 down-regulate the function of HSCs from another aspect.

MAPK pathway is involved in the activation of HSCs and we previously found that knockdown of NR4A2 led to decrease of phosphorylation of MAPK. The Western blotting analysis showed reduced phosphorylation of ERK1/2, P38 and JNK when NR4A2 was knocked down in HSCs^15^. And on the contrary we observed NR4A2 gene over-expression in HSCs caused the increase of phosphorylation of ERK1/2 and P38. Glass *et al*. also reported that nuclear receptors were involved in regulation of signaling pathways and some nuclear receptors could inhibit inflammatory signaling pathways directly in macrophages and T cells^29^. Our results are similar with Glass *et al*’s. And it implied NRA42 may suppress the activation of HSCs via phosphorylation of MAPK pathway.

In addition we validated AdNR4A2 effect in liver fibrosis *in vivo.* Histological examination results showed AdNR4A2 therapy reverted fibrotic degree to stage 2 from stage 3 and decreased the collage content. Fibrosis score analysis indicated that the fibrotic area reduced sharply after AdNR4A2 treatment. We also observed that the NR4A2 protein and mRNA level reduced obviously and α-SMA levels elevated significantly when DMN was applied to induce liver fibrosis in rats. Meanwhile AdNR4A2 therapy gave rise to the increase of NR4A2 protein and mRNA level and the reduction of α-SMA protein and mRNA level. Taken together, AdNR4A2 treatment ameliorated liver fibrosis significantly *in vivo*. At the same time we noticed high expression of NR4A2 in hepatocytes in liver tissue. It is not contradictory as other cells express NR4A2 *in vivo*. Taura *et al*. demonstrated that hepatocytes are not involved in liver fibrogenesis^30^. There were similar reports on NR4A member. Yin *et al*. discovered that the expression of NA4A1, NR4A2 and NR4A3 in leiomyoma was remarkably lower than that in myometrium and the reductions in NA4A2 and NR4A3 were significantly higher than NR4A1^31^. Our investigation is consistent with Yin *et al*. that NR4A2 level in fibrotic liver is lower compared with normal liver tissue. Leuprolide acetate is usually applied for the treatment of uterine fibroid. Yin *et al*. further found that Leuprolide acetate could suppress uterine fibrosis lesion via increasing NR4A2 level. In our study, we raised NR4A2 level through AdNR4A2 treatment *in vivo* and NR4A2 gene over-expression suppressed liver fibrosis in the end. It is evident that Yin *et al*.’s report strongly supports our view.

NR4A2 involved in regulating cell cycle and cell apoptosis^24^. We investigate the NR4A2 effect on apoptosis *in vivo*. Our study indicated that AdNR4A2 treatment increased the apoptosis rate in fibrotic liver. It suggested AdNR4A2 may abate liver fibrosis through promoting cell apoptosis *in vivo*.

In conclusion, our results showed NR4A2 gene over-expression could arrest cell cycle, promote cell apoptosis, deactivate MAPK pathway, weaken migration and invasion ability and reduce collagen production *in vitro*. In animal models AdNR4A2 treatment suppressed liver fibrosis obviously *in vivo*. These results suggest AdNR4A2 exhibit excellent anti-fibrotic effect. NR4A2 may be an ideal gene target for anti-fibrotic therapy and developing some agents increasing NR4A2 expression is very valuable for future clinical application.

## Methods

### Animals and treatments

Male Sprague-Dawley rats (SLAC, Shanghai, China), weighing approximately 250–270 g were maintained in a 12-hour dark/12-hour light cycle. The animal protocol was approved by the Animal Care and Use Committee of Shanghai Jiao Tong University Affiliated Sixth People’s Hospital (License No. SYXK2011-0128). And all methods were performed in accordance with the relevant guidelines and regulations. The liver fibrosis model was induced by injection of DMN as described previously^32^. Normal group served as control which received intraperitoneal saline injection. Other group rats were injected intraperitoneally with 1% DMN (10 ug/kg; Sigma, St.Louis, USA) for 3 consecutive days per week up to 4 weeks and infused with phosphate-buffered saline (model group), 5 × 10^9^ pfu AdNR4A2 (AdNR4A2 group) or AdNC (AdNR4A2 group) via the tail vein respectively after six repeated DMN injection. The animals were sacrificed 4 weeks after DMN administration.

### Adenovirus and cell lines

The HSC-T6 cells were kindly provided by Dr. Friedman of Mount Sinai School of Medicine of New York University (MSSM). Adenovirus targeting NR4A2 and its negative control (GenePharma, Shanghai, China) infected primary hepatic stellate cells or HSC-T6 cells at a multiplicity of infection of 40 for 72 hour. Cells were cultured in DMEM medium supplemented with 10% heat-inactivated fetal calf serum (FBS) (Gibco NY, USA). All cells were maintained in a humidified incubator at 37 °C with 5% CO2.

### Real time-polymerase chain reaction

Total RNA was purified from HSC cells or liver using Trizol (Takara, Dalian, China) and reverse transcribed was performed using PrimeScript RT Master Mix (Takara). Real time polymerase chain reaction (RT-PCR) was conducted by using SYBR Green PCR Kit (Takara) and Applied Biosystems 7500 real-time PCR system (Applied Biosystems, Foster City, CA, USA). Primer sequences (Sangon Biotech, Shanghai, China) were as follows: NR4A2 (Fw: 5′-AGATTCCTGGCTTTGCTGAC-3′; Rev: 5′-CTGGGTTGGACCTGTATGCT-3′); α-SMA (Fw: 5′-CCAGGGAGTGATGGTTGGA-3′; Rev: 5′-CCGTTAGCAAGGTCGGATG-3′); TGF-β (Fw: 5′-TGGCGTTACCTTGGTAACC-3′; Rev: 5′-GGTGTTGAGCCCTTTCCAG-3′); Col I (Fw: 5′-AGGCATAAAGGGTCATCGTG-3′; Rev:5′-ACCGTTGAGTCCATCTTTGC-3′); Col III (Fw: 5′-AGGCCAATGGCAATGTAAAG-3′; Rev:5′-TATTGGTGGGTGAAACAGCA-3′); β-actin (Fw: 5′-ACCCACACTGTGCCCATCTATG-3′; Rev: 5′-AGAGTACTTGCGCTCAGGAGGA-3′). The resulting sequences were normalized to the β-actin expression levels, and relative gene expression was meas-ured by the 2^−ΔΔCT^ method.

### Immunohistochemistry and immunofluorescence

Hepatic tissues were embedded in paraffin and sectioned. The sections were incubated in 3% H2O2 in methanol and nonspecific binding was blocked with 10% normal goat serum. The sections were incubated with primary antibody overnight, washed and incubated with secondary antibody for 60 minutes. Antigen–antibody complexes were visualized using DAB kits (GeneTech, Shanghai, China). Immunofluorescence was performed as described previously^33^. The following primary antibodies were used: α-smooth muscle actin (α-SMA) (Santa Cruz), NR4A2 (Santa Cruz), Alexa Fluor^®^ 546 IgG (Life Technologies), Alexa Fluor^®^ 488 IgG (H + L) (Life Technologies). Image scanning analysis system (Image-Pro Plus) was used to analyze the changes in integrated optical density (IOD) of NR4A2 and α-SMA in immunofluorescence images.

### Primary HSC isolation

Sprague-Dawley rats (SLAC, Shanghai, China) for primary HSCs were prepared. After anesthesia, the rat abdominal wall was opened and the intestines were moved aside so as to expose the vena cava and portal vein. A fine cannula ligated and fixed to a proper location was inserted into the vein after placing a thread around it. Perfusion solutions were incubated at 37 °C before usage. An adjustable peristaltic pump was then used to control the *in situ* liver perfusion process. After the liver swelled, the cannula in the inferior vena cava was opened to allow draining. Subsequently, the liver was perfused in turn with D-HanK’s solution, Collagenase IV solution, Pronase solution for 15 min at 37 °C. The cell fraction was collected, washed and put between a top layer of buffer and a bottom cushion of 18% Nycodenz. After centrifugation at 1400 g for 22 minutes, the HSCs fraction at the interface between the top and intermediate layer was obtained. Finally, the cells were cultured in DMEM medium supplemented with 10% FBS.

### Western blot

Western blot analysis was performed as described previously^34^. Western blots were performed using antibodies against GAPDH (Santa Cruz), IRDye680 anti-rabbit (Life Technologies), IRDye680 anti-mouse (Life Technologies), Erk1/2 (Cell Signaling, MA, USA), P-erk1/2 (Cell Signaling), P-P38 (Cell Signaling), P38(Cell Signaling). Protein samples were transferred onto a polyvinylidenefluoride membrane and were scanned through Odyssey Infrared Imaging System.

### Measurement of Hepatic Hydroxyproline Content

Total hepatic hydroxyproline levels Western blot analysis was determined according to standard protocols in the hydrolysates of liver samples as described^32^.

### Cell cycle analysis

Cells were seeded into 6-well cell culture plates at a density of 5 × 10^5^ cells. The cells were transfected with AdNR4A2 or AdNC respectively and 72 hour later the cells were harvested, stained and fixed with propidium iodide (PI) according to the manufacturer’s protocol and subject to cell cycle analysis using flow cytometer (BD, NK, USA).

### Cell apoptotic assay

Cells were seeded into 6-well cell culture plates at a density of 5 × 10^5^ cells. The cells were transfected with AdNR4A2 or AdNC respectively and 72 hours later the cells were harvested. Apoptosis assay was measured using FITC AnnexinV Apoptosis Detection Kit (Keygen, NJ, China) according to the manufacturer’s protocol and subject to cell cycle analysis using flow cytometer.

### Transwell migration assay

Transwell chambers (BD, Franklin Lakes, NJ, USA) were set up with 8-mm pore size filters to assess migration ability. HSC-T6 cells were harvested at the logarithmic phase and infected with AdNR4A2 or AdNC respectively for 72 hour. Then cells were harvested and 0.2 × 10^5^ cells were seeded onto the chamber’s upper compartment with 200 μl of serum-free medium and each group had 3 holes. At the same time, 500 μl medium with 10% serum was added into the lower chamber. After 8 hour incubation at 37 °C and 5% CO2 culture, the migrated cells would locate on the lower surface of the transwell filters. Then, these cells were fixed for 30 min in methanol and stained with 0.1% crystal violet for another 30 min. In each of 3 randomly selected sights under the microscope magnifying 200, the stained cells were observed and counted.

### Histological Examination

All paraffin-embedded liver tissues were stained with hematoxylin and eosin, Sirius red staining and Masson’s trichrome. Connective tissues stained blue with Masson’s trichrome were measured on an image analyzer (Image J, National Institutes of Health). Three fields were selected randomly and six rats from each group were examined. The percentage was calculated as described previously^35^.

### Statistical analysis

The results were expressed as mean ± SD and were analyzed by analysis of variance (ANOVA) or Student’s t-test followed by paired comparison as appropriate. P < 0.05 was taken as the minimum level of significance.

## Additional Information

**How to cite this article**: Chen, P. *et al*. Adenovirus-mediated expression of orphan nuclear receptor NR4A2 targeting hepatic stellate cell attenuates liver fibrosis in rats. *Sci. Rep.* **6**, 33593; doi: 10.1038/srep33593 (2016).

## Supplementary Material

Supplementary Dataset 1

Supplementary Dataset 2

## References

[b1] Schuppan D. Structure of the extracellular matrix in normal and fibrotic liver: collagens and glycoproteins. Semin Liver Dis 10, 1–10 (1990). 2186485 10.1055/s-2008-1040452

[b2] Wake K. Perisinusoidal stellate cells (fat-storingcells, interstitial cells, lipocytes), their related structure in and around the liver sinusoids and vitaminA-storing cells in extra hepatic organs. Int Rev Cytol 66, 303–353 (1980). 6993411 10.1016/s0074-7696(08)61977-4

[b3] Choi S. S. & Diehl A. M. Epithelial-to-mesenchymal transitions in the liver. Hepatology 50, 2007–2013 (2009). 19824076 10.1002/hep.23196 PMC2787916

[b4] Okada H., Strutz F., Danoff T. M., Kalluri R. & Neilson E. G. Possible mechanisms of renal fibrosis. Contrib Nephrol 118, 147–154 (1996). 8744052 10.1159/000425088

[b5] Lee J. M., Dedhar S., Kalluri R. & Thompson E. W. The epithelial-mese-nchymal transition: new insights insignaling, development and disease. J Cell Biol 172, 973–981 (2006). 16567498 10.1083/jcb.200601018 PMC2063755

[b6] Mederacke I. . Fate tracing reveals hepatic stellate cells as dominant contributors to liver fibrosis in dependent of its aetiology. Nat Commun 4, 2823 (2013). 24264436 10.1038/ncomms3823 PMC4059406

[b7] Geerts A. History, heterogeneity, developmental biology and functions of quiescent hepatic stellate cells. Semin Liver Dis 21, 311–335 (2001). 11586463 10.1055/s-2001-17550

[b8] Friedman S. L. Hepatic stellate cells:protean, multifunctional, and enigmatic cells of the liver. Physiol Rev 88, 125–172 (2008). 18195085 10.1152/physrev.00013.2007 PMC2888531

[b9] Friedman S. L., Roll F. J., Boyles J. & Bissell D. M. Hepatic lipocytes:the principal collagen-producing cells of normal rat liver. Proc Natl Acad Sci USA 82, 8681–8685 (1985). 3909149 10.1073/pnas.82.24.8681 PMC391500

[b10] Ramachandran P. & Iredale J. P. Reversibility of liver fibrosis. Ann.Hepatol 8, 283–291 (2009). 20009126

[b11] Henderson N. C. & Iredale J. P. Liver fibrosis:cellular mechanisms of progression and resolution. Clin Sci 112, 265–280 (2007). 17261089 10.1042/CS20060242

[b12] Mallet V. . Brief communication: The relationship of regression of cirrhosis to outcome in chronic hepatitis C. Ann Intern Med 149, 399–403 (2008). 18794559 10.7326/0003-4819-149-6-200809160-00006

[b13] Friedman S. L. & Bansal M. B. Reversal of hepatic fibrosis—factor fantasy? Hepatology 43, S82–S88 (2006). 16447275 10.1002/hep.20974

[b14] Bansal R. . Engineered targeted interferon-gamma blocks hepatic fibrogenesis in mice. Hepatology 54, 586–596 (2011). 21538439 10.1002/hep.24395

[b15] Chen P. . Orphan nuclear receptor NR4A2 inhibits hepatic stellate cell proliferation through MAPK pathway in liver fibrosis. PeerJ 3, e1518 (2015). 26713258 10.7717/peerj.1518 PMC4690364

[b16] Davis B. H. & Vucic A. The effect of retinol on Ito cell proliferation *in vitro*. Hepatology 8, 788–793 (1988). 3391506 10.1002/hep.1840080416

[b17] Hernandez-Gea V. & Friedman S. L. Pathogenesis of liver fibrosis. Annu Rev Pathol 6, 425–456 (2011). 21073339 10.1146/annurev-pathol-011110-130246

[b18] Martinez-Hernandez A. & Amenta P. S. The extracellular matrix in hepatic regeneration. FASEB J 9, 1401–1410 (1995). 7589981 10.1096/fasebj.9.14.7589981

[b19] Saxena N. K. . Concomitant activation of the JAK/STAT, PI3K/AKT, and ERK signaling is involved in leptin-mediated promotion of invasion and migration of hepatocellular carcinoma cells. Cancer Res 67, 2497–2507 (2007). 17363567 10.1158/0008-5472.CAN-06-3075 PMC2925446

[b20] Deutsch A. J., Angerer H., Fuchs T. E. & Neumeister P. The Nuclear Orphan Receptors NR4A as Therapeutic Target in Cancer Therapy. Anti-Cancer Agents in Medicinal Chemistry 12, 1001–1014 (2012). 22583411 10.2174/187152012803529619

[b21] Hellemans K. . Differential modulation of rat heaptic stellate phenotype by natural and synthetic retinoids. Hepatology 39, 97–108 (2004). 14752828 10.1002/hep.20015

[b22] Chen H., He Y. W., Liu W. Q. & Zhang J. H. Rosiglitazone prevents murine Hepatic fibrosis induced by Schistosomajaponicum. World J Gastroenterol 14, 2905–2911 (2008). 18473419 10.3748/wjg.14.2905 PMC2710736

[b23] Yang L., Stimpson S. A., Chen L., Wallace Harrington W. & Rockey D. C. Effectiveness of the PPAR-gamma agonist, GW570, in liver fibrosis. Inflamm Res 59, 1061–1071 (2010). 20585829 10.1007/s00011-010-0226-0

[b24] Zhao D., Desai S. & Zeng H. VEGF stimulates PKD-mediated CREB-dependent Orphan nuclear receptor Nurr1 expression: role in VEGF-induced angiogenesis. International Journal of Cancer 128, 2602–2612 (2011). 20715116 10.1002/ijc.25600

[b25] Li H. . Cytochrome c release and apoptosis induced by mitochondrial targeting of nuclear orphan receptor TR3. Science 289, 1159–1164 (2000). 10947977 10.1126/science.289.5482.1159

[b26] Moll U. M., Marchenko N. & Zhang X. K. p53 and Nur77/TR3 -transcription factors that directly target mitochondria for cell death induction. Oncogene 25, 4725–4743 (2006). 16892086 10.1038/sj.onc.1209601

[b27] Thompson J. & Winoto A. During negative selection, Nur77 family proteins translocate to mitochondria where they associate with Bcl-2 and expose its proapoptotic BH3 domain. J Exp Med 205, 1029–1036 (2008). 18443228 10.1084/jem.20080101 PMC2373836

[b28] Friedman S. L. Evolving challenges in hepatic fibrosis. Nat Rev Gastroenterol Hepatol 7, 425–436 (2010). 20585339 10.1038/nrgastro.2010.97

[b29] Glass C. K. & Saijo K. Nuclear receptor transrepression pathways that regulate inflammation in macrophages and T cells. Nat Rev Immunol 10, 365–376 (2010). 20414208 10.1038/nri2748

[b30] Taura K. . Hepatocytes do not undergo epithelial-mesenchymal transition in liver fibrosis in mice. Hepatology 51, 1027–1036 (2010). 20052656 10.1002/hep.23368 PMC2906231

[b31] Yin H. . Expression profiling of nuclear receptors identifies key roles of NR4A subfamily in Uterine fibroids. Molecular Endocrinology 27, 726–740 (2013). 23550059 10.1210/me.2012-1305 PMC3634117

[b32] Lin Y. . Treatment of experimental hepatic fibrosis by combinational delivery of urokinase-type plasminogen activator and hepatocyte growth factor genes. Liver Int 25, 796–807 (2005). 15998431 10.1111/j.1478-3231.2005.01098.x

[b33] García-Pérez D. . Morphine regulates Argonaute2 and TH expression and activity but not miR-133b in Midbrain dopaminergic neurons. Addict Biol 20, 104–119 (2015). 23927484 10.1111/adb.12083

[b34] Ogawa T. . MicroRNA-221/222 upregulation indicates the activation of stellate cells and the progression of liver fibrosis. Gut 61, 1600–1609 (2012). 22267590 10.1136/gutjnl-2011-300717

[b35] Guimond A. . Quantitative ultrasonic tissue characterization as a new tool for continuous monitoring of chronic liver remodeling in mice. Liver Int 27, 854–864 (2007). 17617129 10.1111/j.1478-3231.2007.01493.x

