# Supplementary material for: Adenovirus-mediated expression of orphan nuclear receptor NR4A2 targeting hepatic stellate cell attenuates liver fibrosis in rats
Source: Sci Rep. 2016 Sep 20;6:33593. doi: 10.1038/srep33593 (PMC5028713; doi:10.1038/srep33593)
Supplement: Supplementary Dataset 1 [file srep33593-s1.docx]

**Adenovirus-mediated expression of orphan nuclear receptor NR4A2 targeting hepatic stellate cell attenuates liver fibrosis in rats**

Pengguo Chen^1,2,3^ , Jie Li^1^ , Yan Huo ^1^, Jin Lu ^1^,Lili Wan^1^ , Quanjun Yang^1^ , Jinlu Huang ^1^, Run Gan^1^ and Cheng Guo^1,2^*

^1^Department of Pharmacy, Shanghai Jiao Tong University Affiliated Sixth People’s Hospital, 600 Yishan Road, Shanghai, China

^2^ Shanghai Jiao Tong University School of Medicine, Shanghai, China

^3^Department of Gastroenterology, Jiangxi Provincial People’s Hospital, 92 Aiguo Road, Nanchang, Jiangxi, China.

*Corresponding author: Department of Pharmacy, Shanghai Jiao Tong University Affiliated Sixth People’s Hospital, 600 Yishan Road, Shanghai 200233, China. Fax: +86 02124058789

*Email adress*: [guopharm@126.com](mailto:guopharm@126.com) (C.Guo)

**A**

**B**

**C****D**
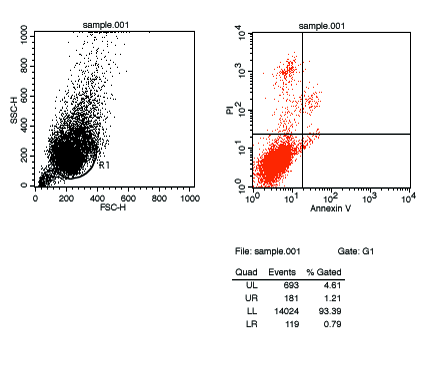


**E**
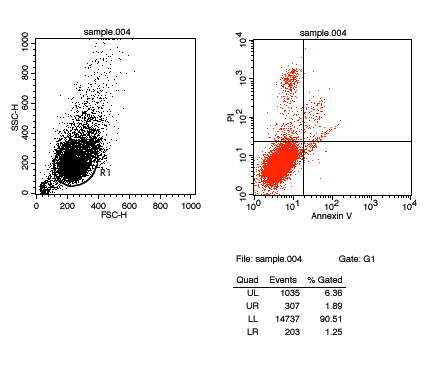


**F**
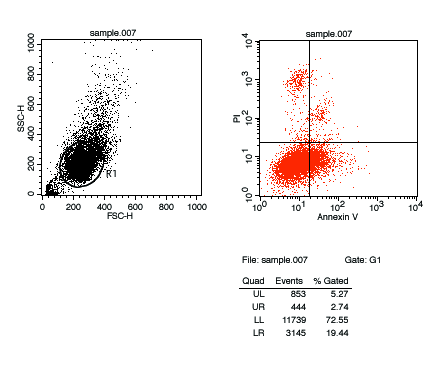


**Figure 1.** AdNR4A2 treatment causes cell cycle arrest and increased cell apoptosis rate. HSC-T6 cells treated with AdNR4A2 and AdNC respectively at an MOI of 40 for 72 hours were stained with propidium iodide and analyzed by FACS. Representative cell cycle flow cytometry graph for control (A), AdNC (B) and AdNR4A2 group (C). Representative apoptosis graph for control (D), AdNC (E) and AdNR4A2 group (F).

**A**
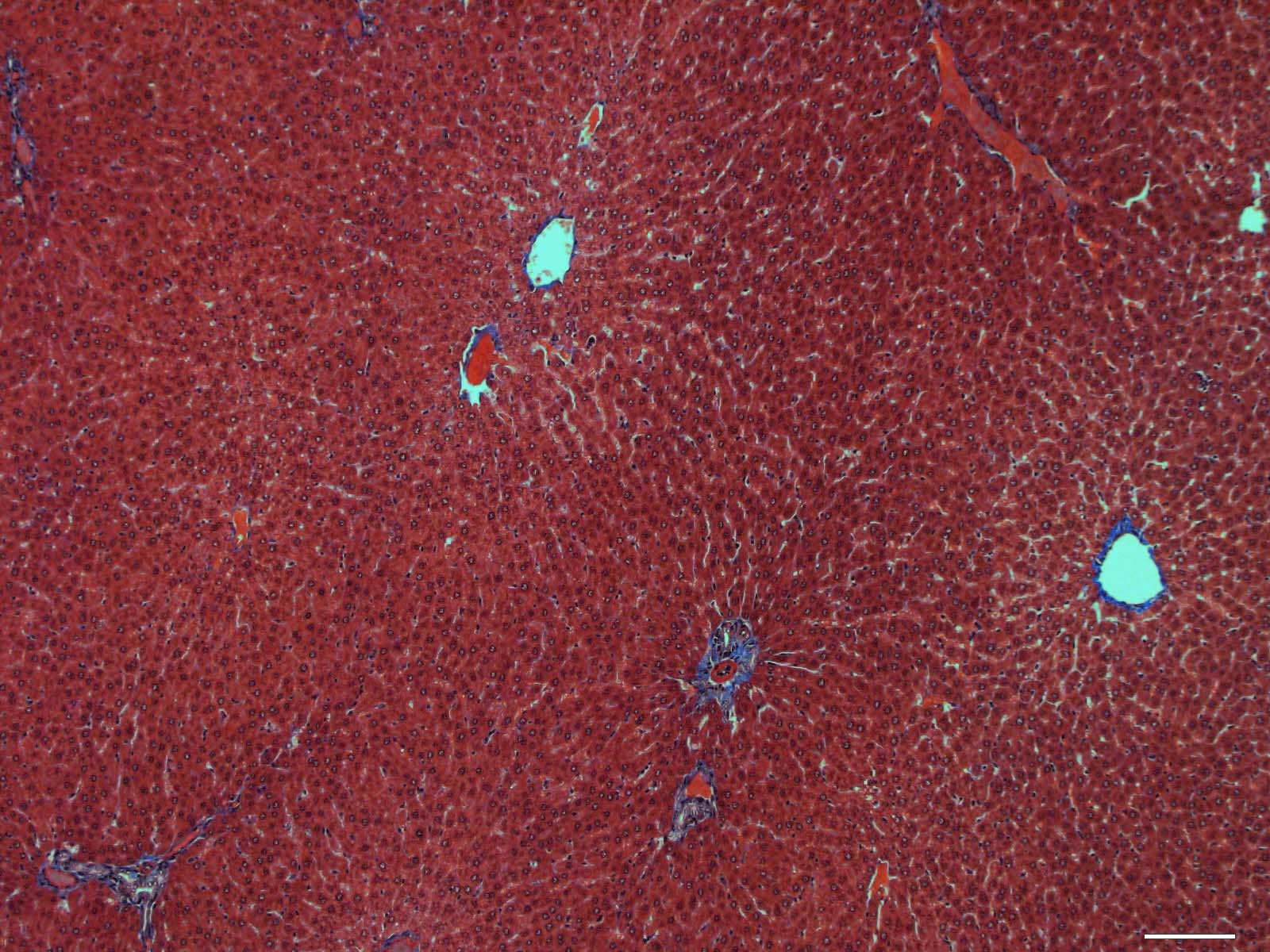


**B**


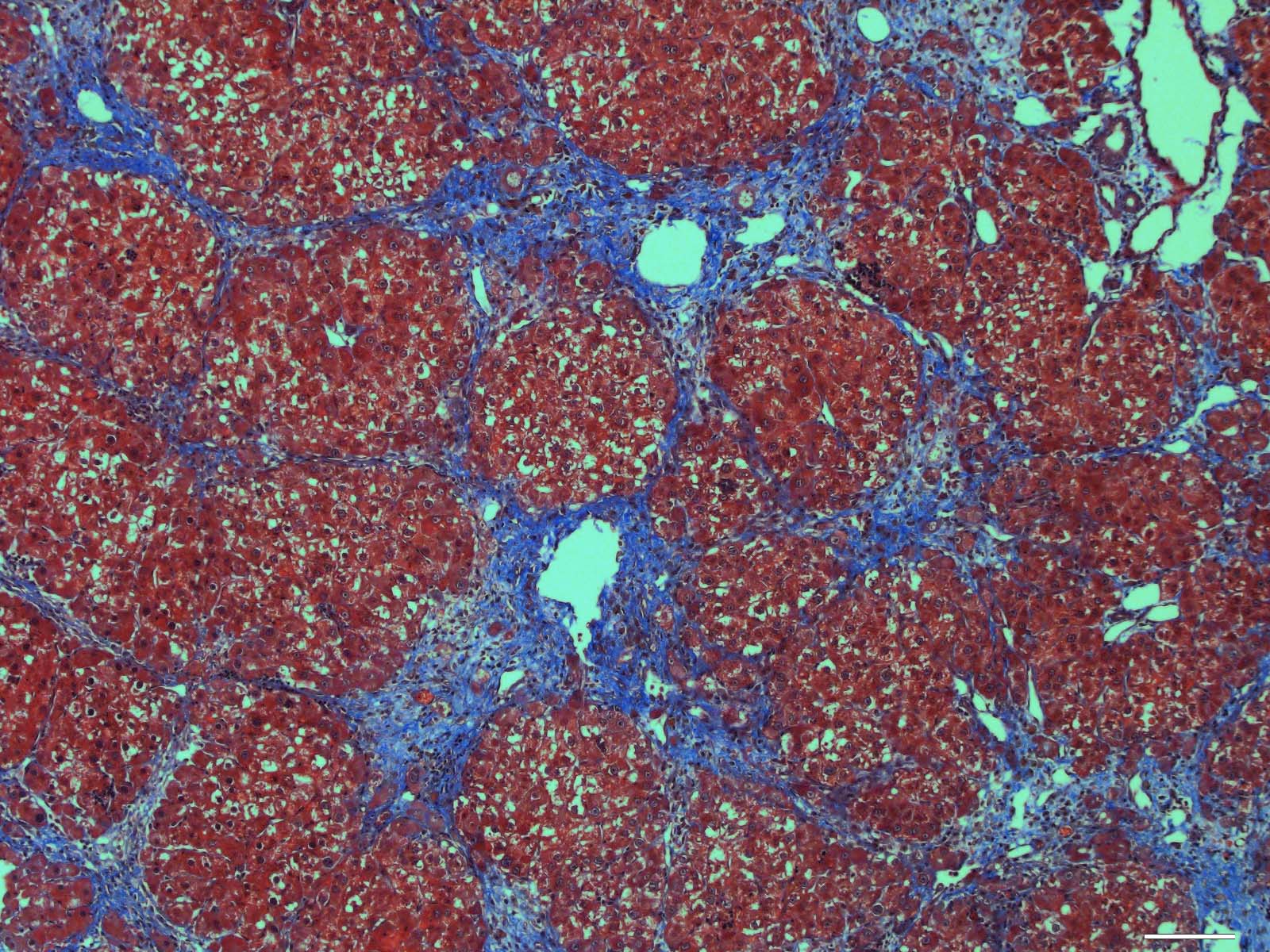


**C**


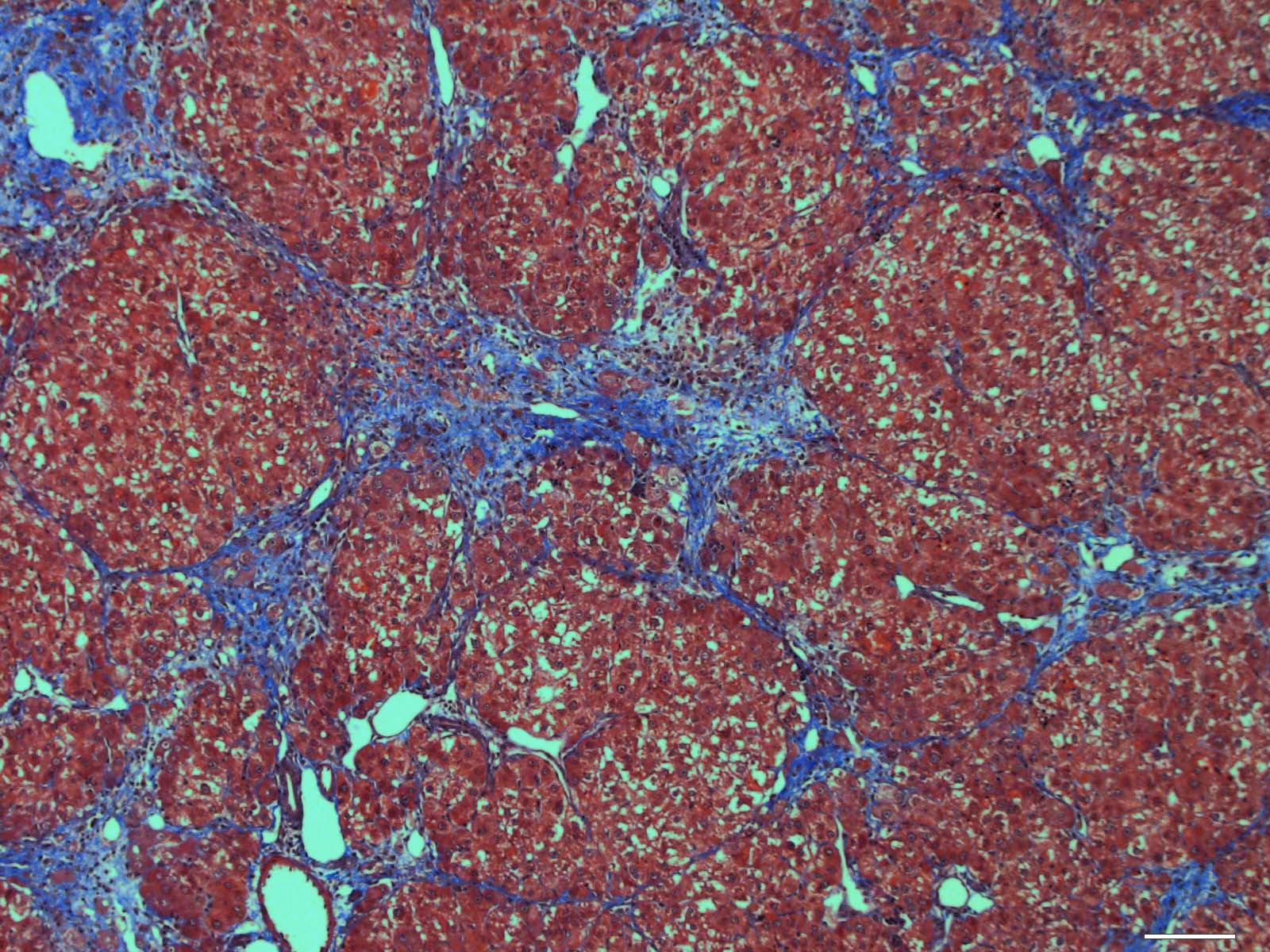


**D**


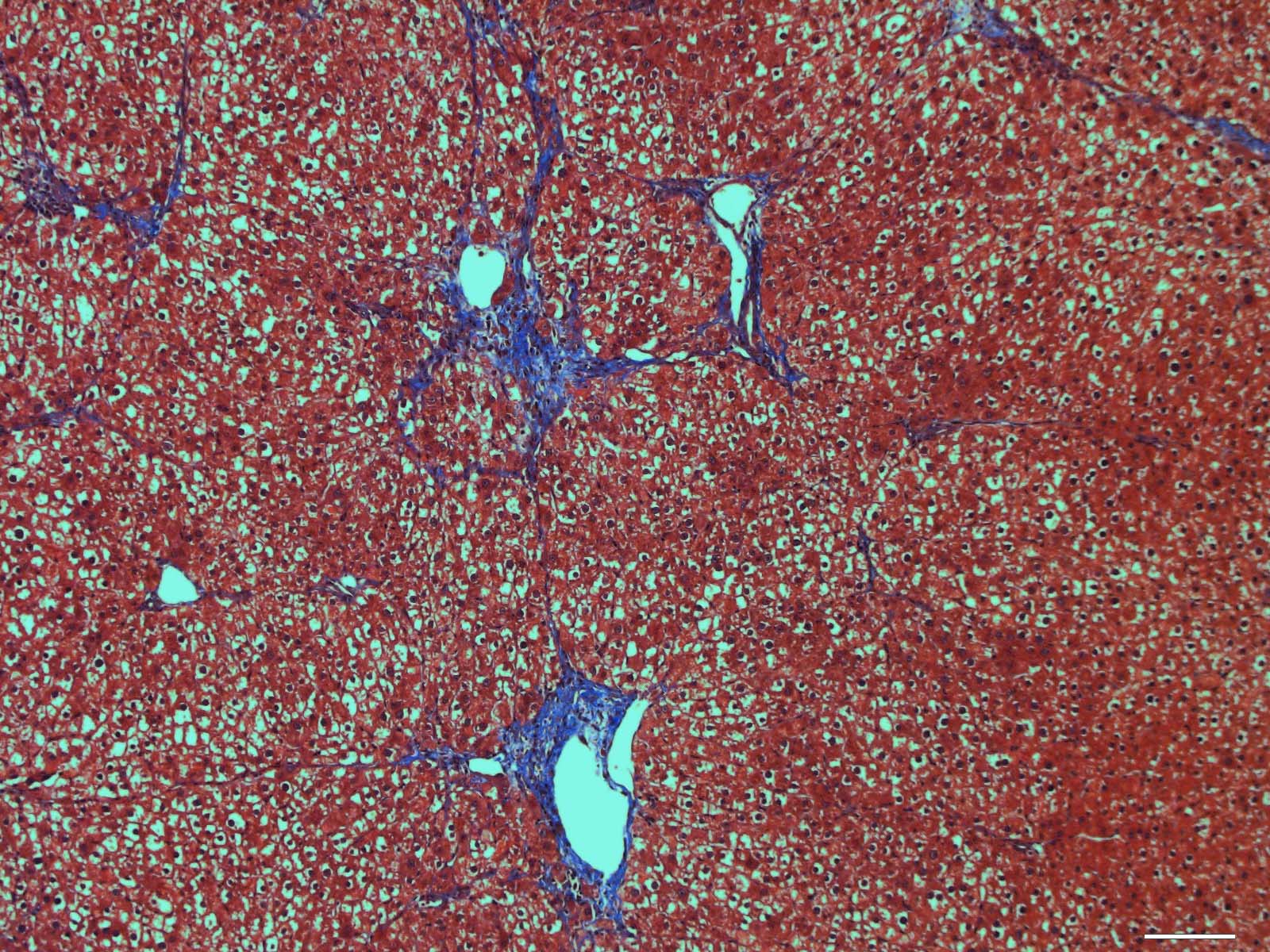


**E**


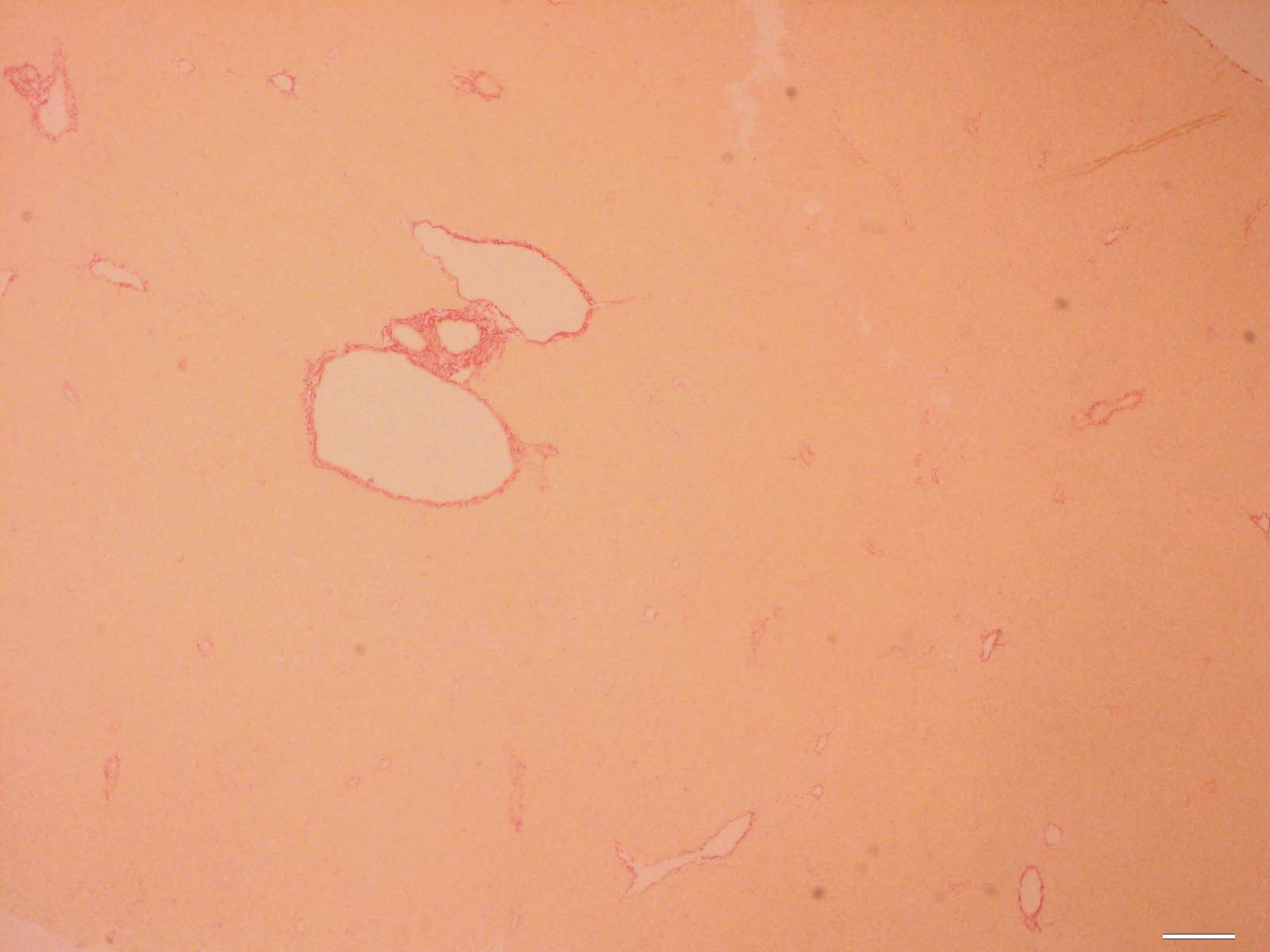


**F**


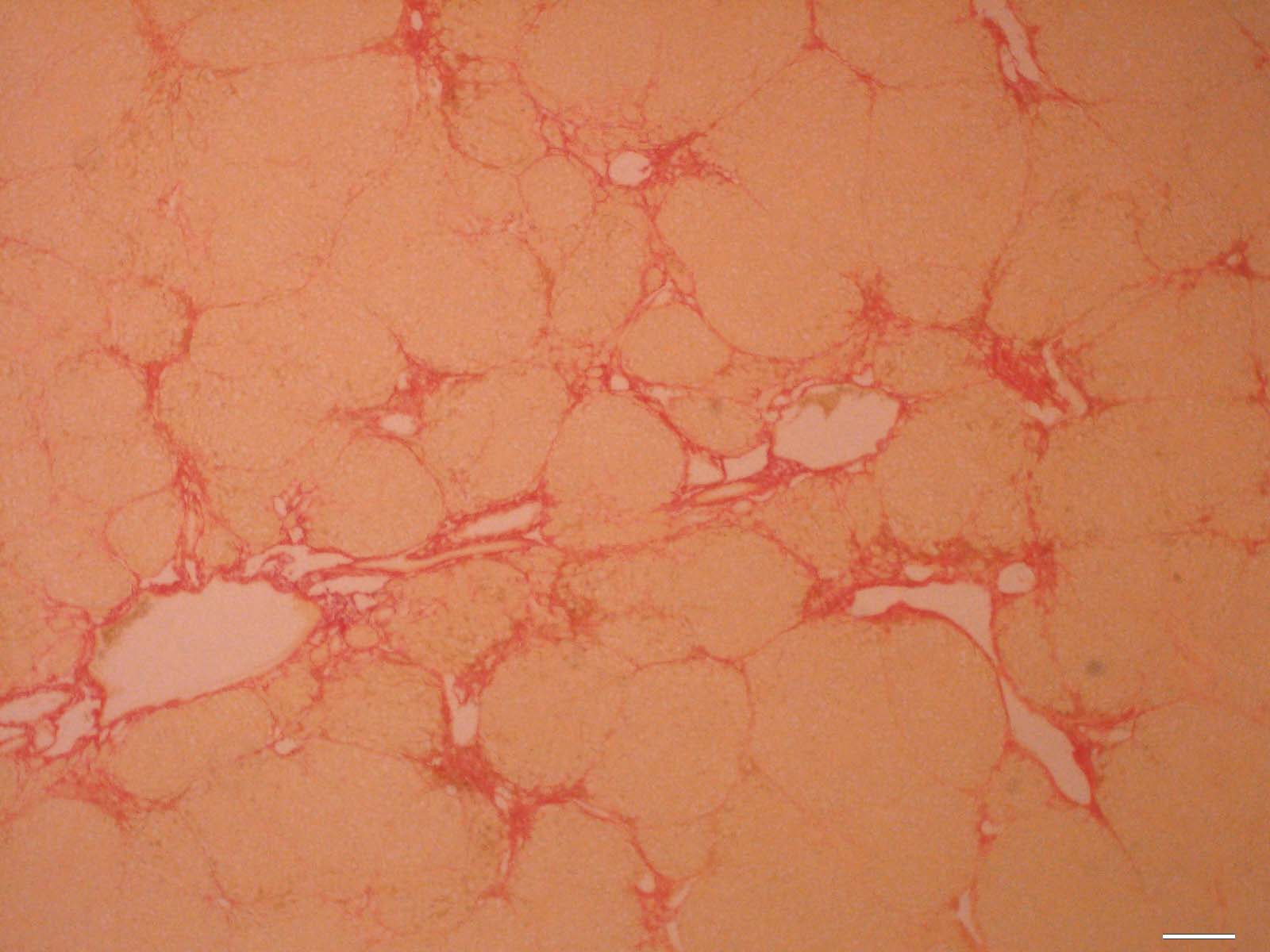


**G**
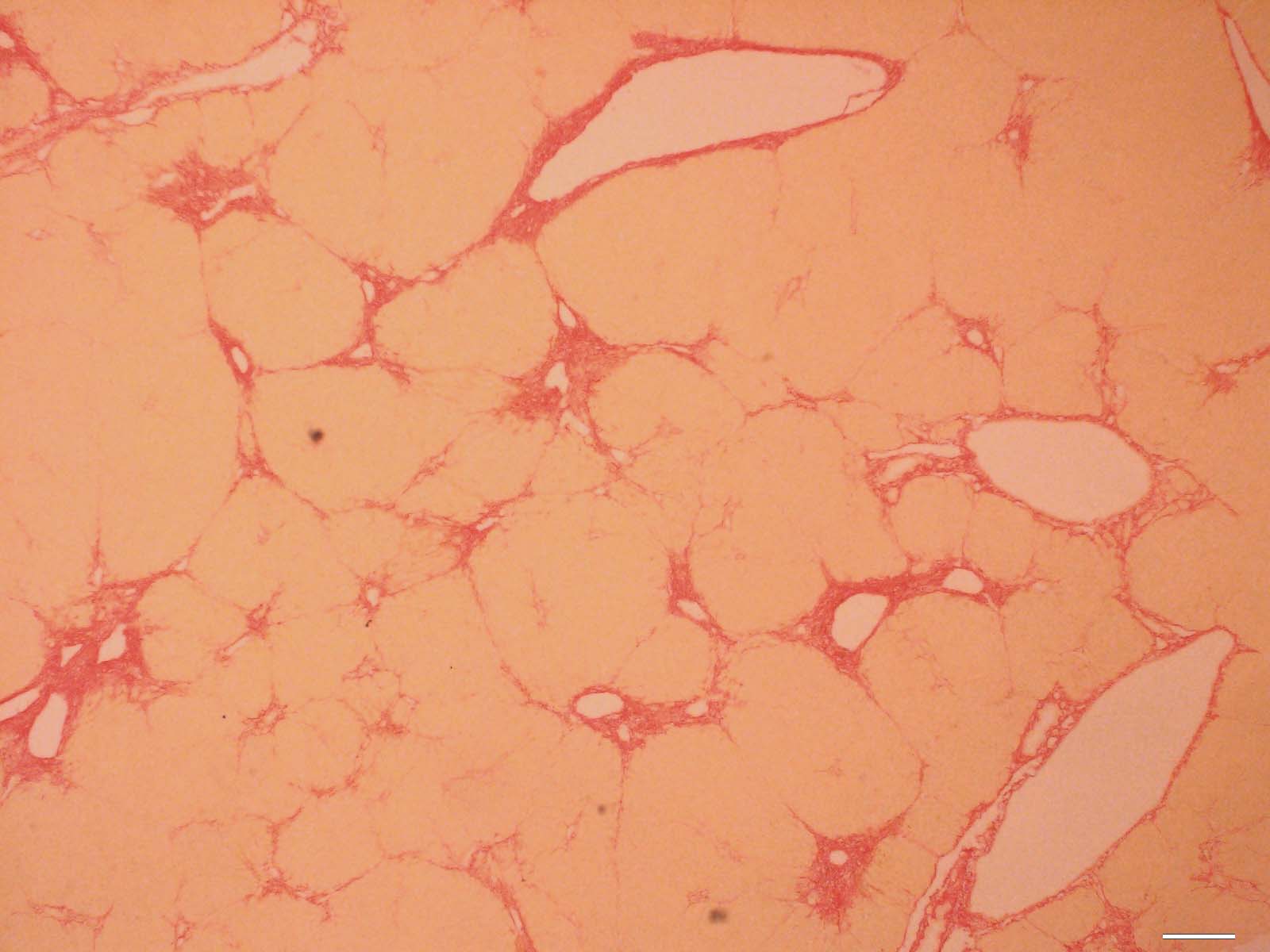


**H**


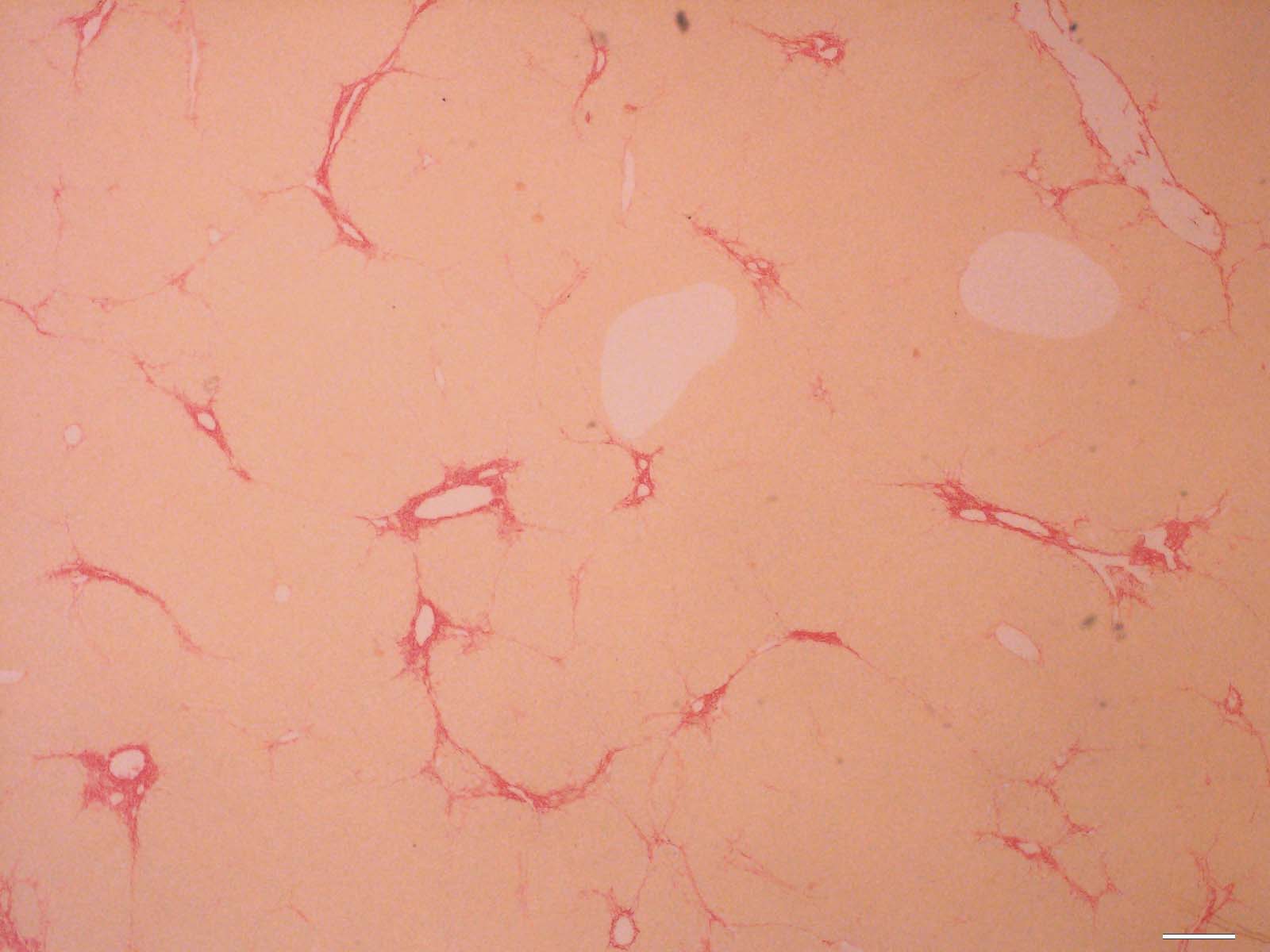


**I**


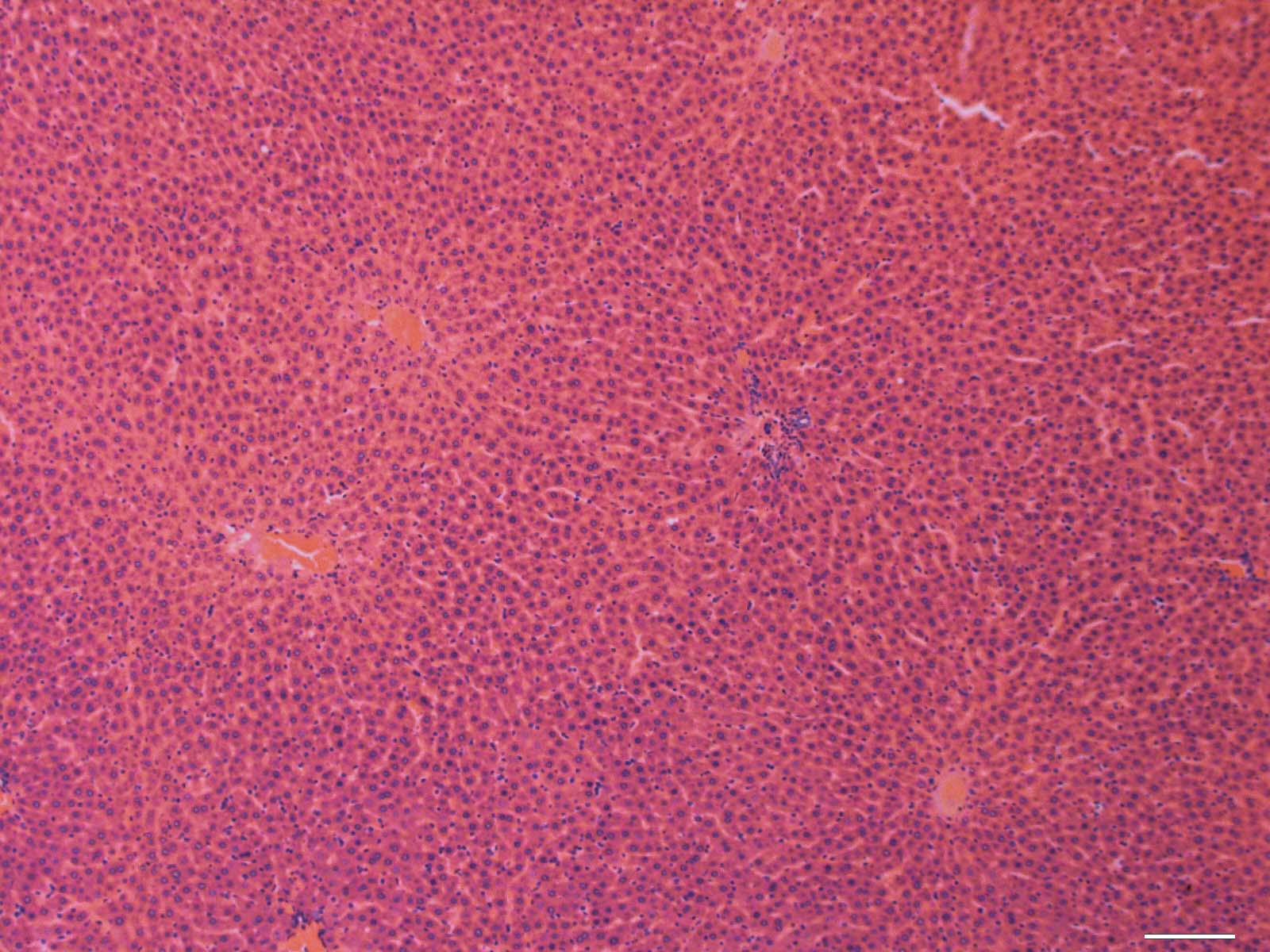


**J**


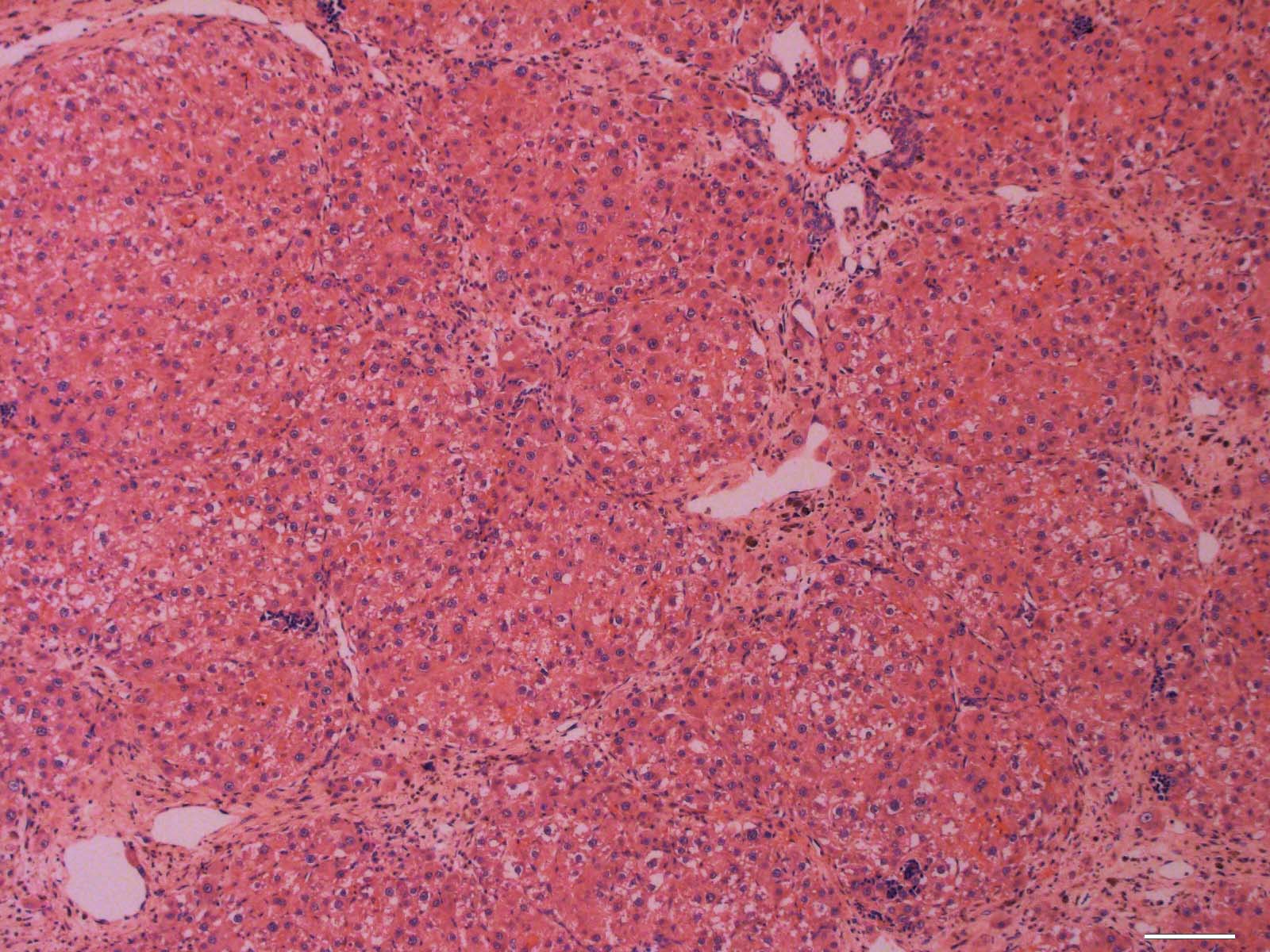


**K**


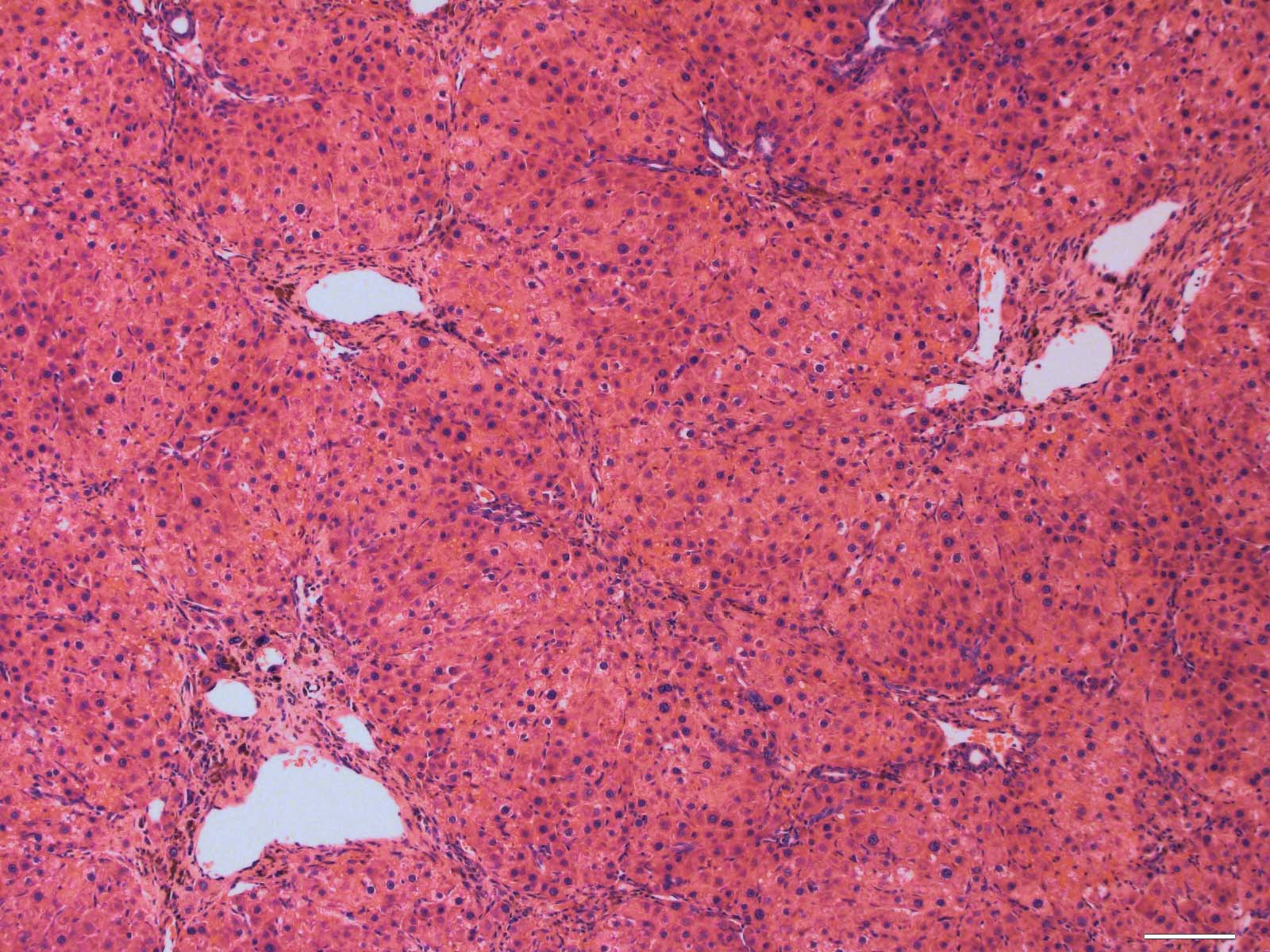


**L**


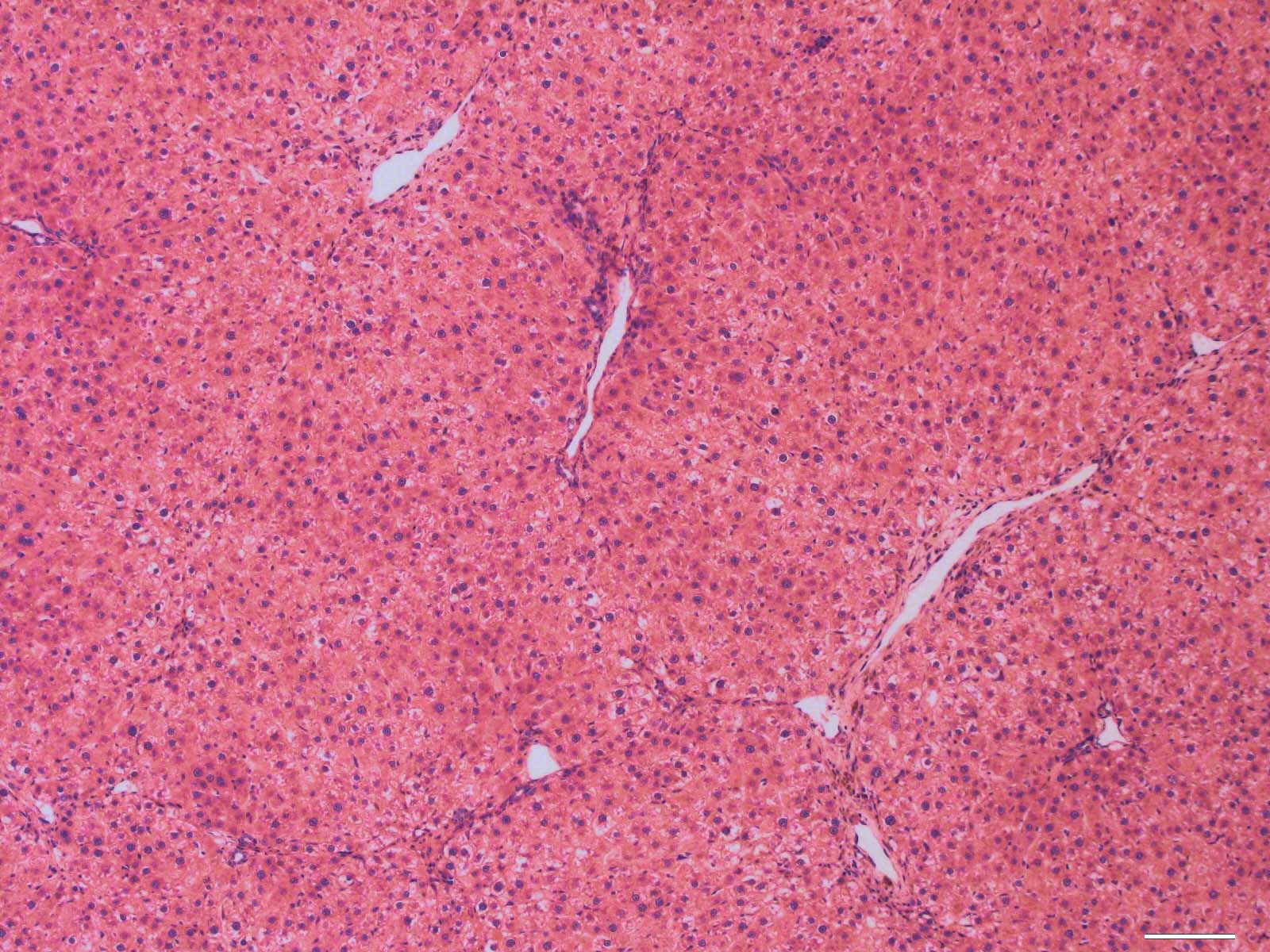


**Figure 2.** AdNR4A2 treatment alleviates dimethyl nitrosamine-induced liver fibrosis. Rats harboring dimethyl nitrosamine-induced hepatic fibrosis were treated by infusion of AdNR4A2, AdNC and medium respectively and sacrificed. The normal healthy rats were also sacrificed meantime. Paraffin-embedded liver sections were visualized by H/E staining, Sirius Red and Masson’s trichrome staining and representative fields of view were photographed at ×4 or ×10 magnification. Masson’s trichrome staining representative images for normal group (A), model group (B), AdNC group (C) and AdNR4A2 group (D). Sirius Red staining representative images for normal group (E), model group (F), AdNC group (G) and AdNR4A2 group (H). H/E staining representative images for normal group (I), model group (J), AdNC group (K) and AdNR4A2 group (L).

**A**


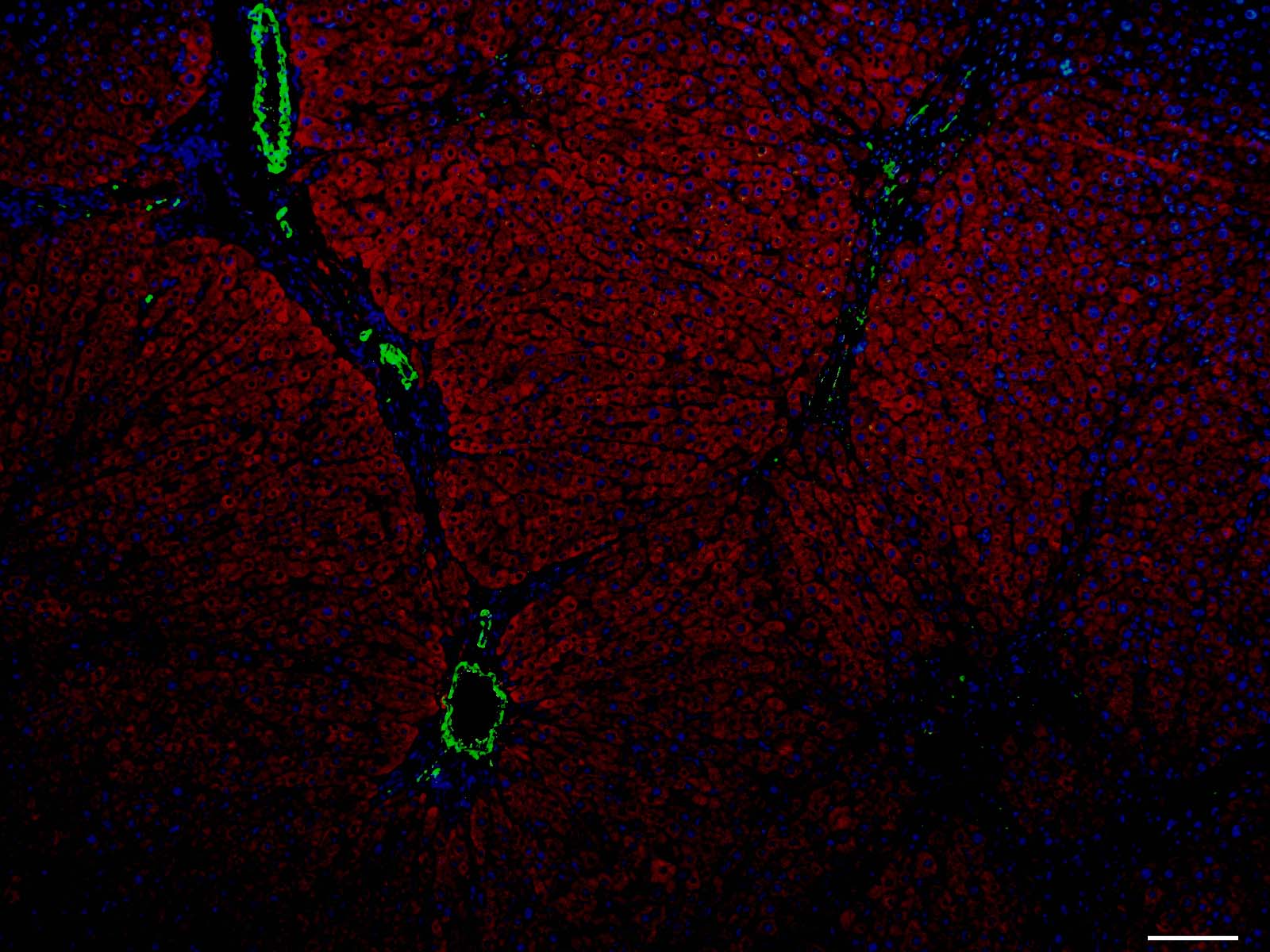


**B**


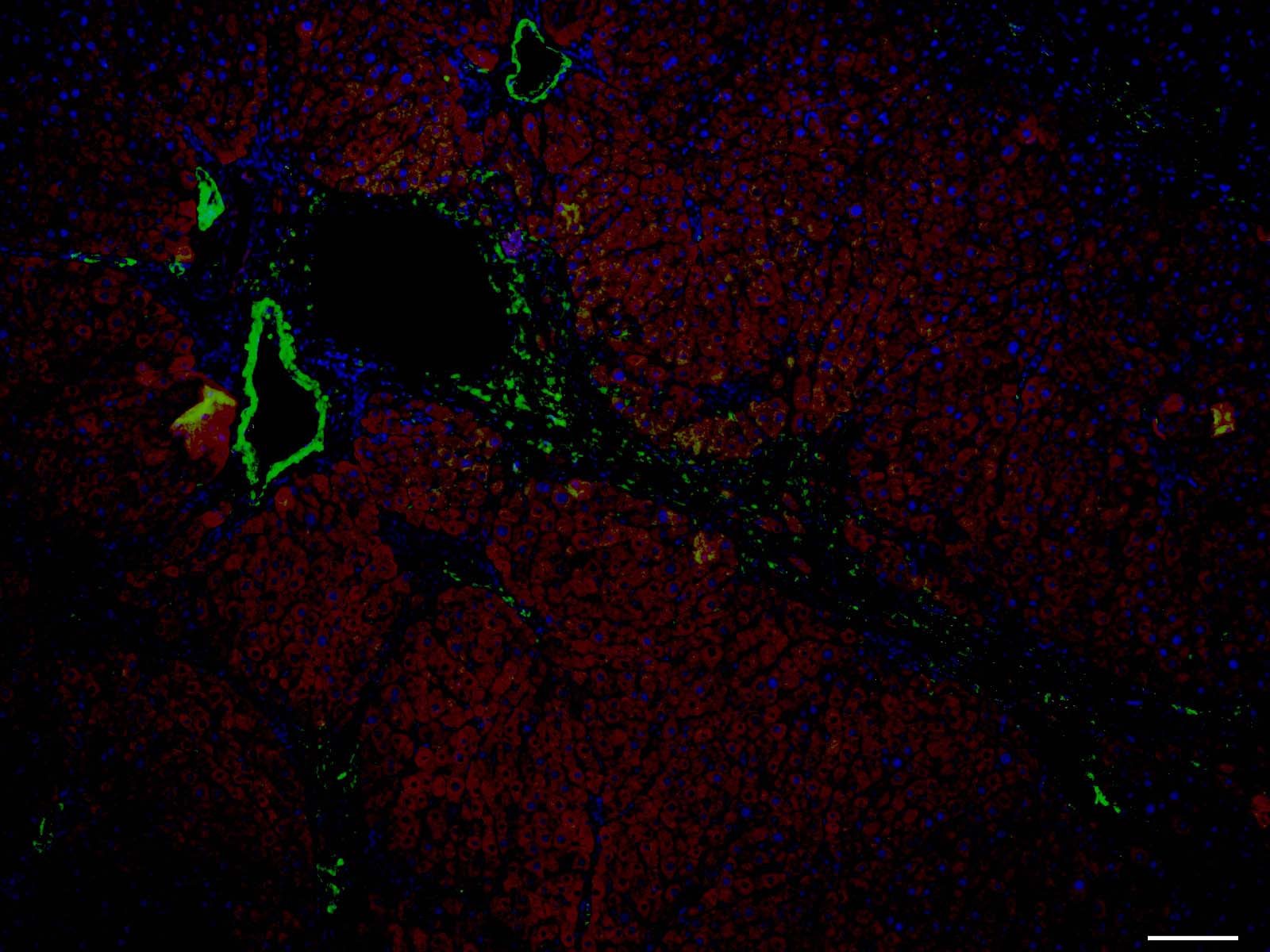


**C**


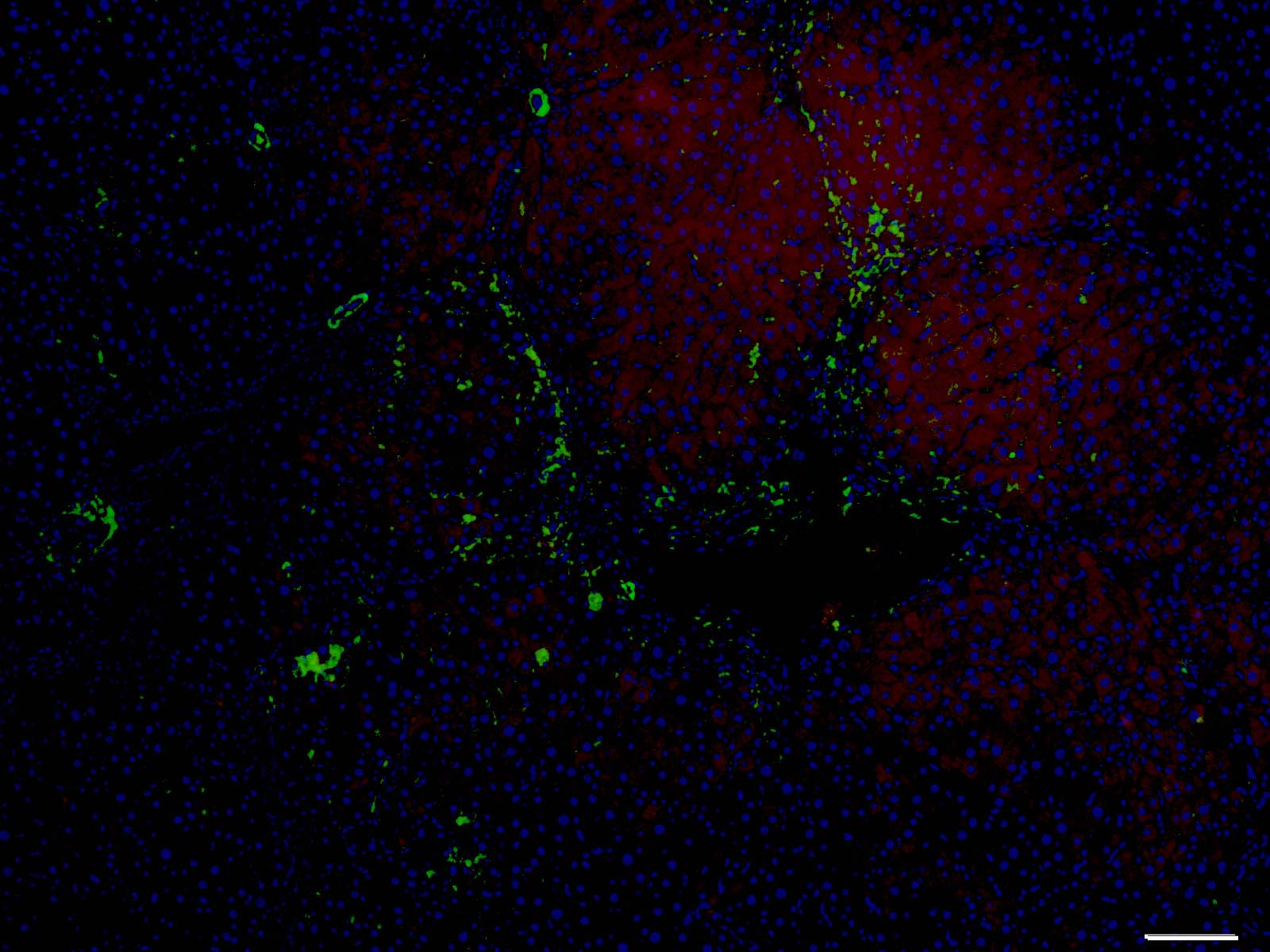


**D**


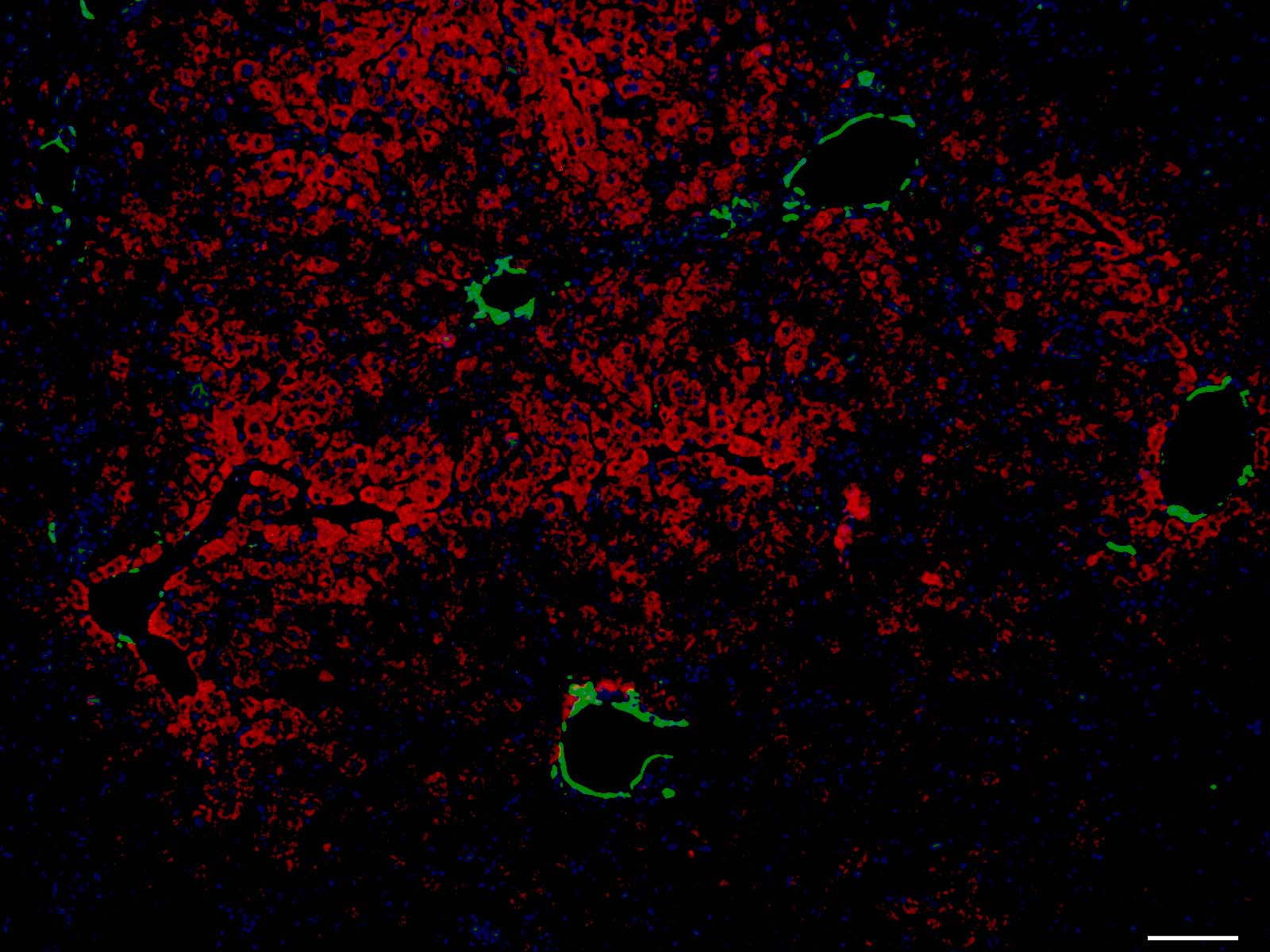


**Figure 3.** AdNR4A2 treatment increases NR4A2 level and decreases α-SMA level in dimethyl nitrosamine-induced fibrotic liver tissue. Rats harboring dimethyl nitrosamine-induced hepatic fibrosis were treated by infusion of AdNR4A2, AdNC and medium respectively and sacrificed. The normal healthy rats were also sacrificed meantime. Paraffin-embedded liver sections were made. Immunofuorescent costaining for NR4A2 (red), α-SMA (green) and DAPI (blue). The representative image for normal group (A), model group (B), AdNC group (C) and AdNR4A2 group (D). scale bar 200 µm.

**A**


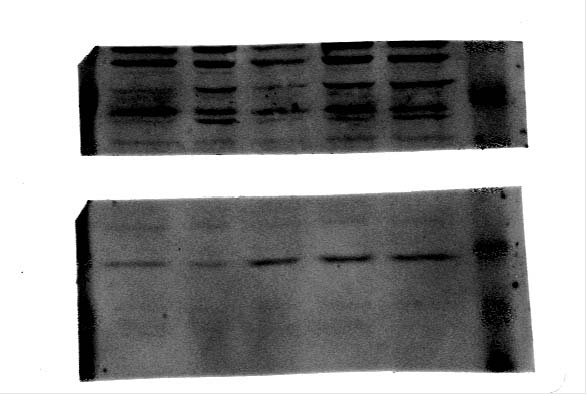


**B**


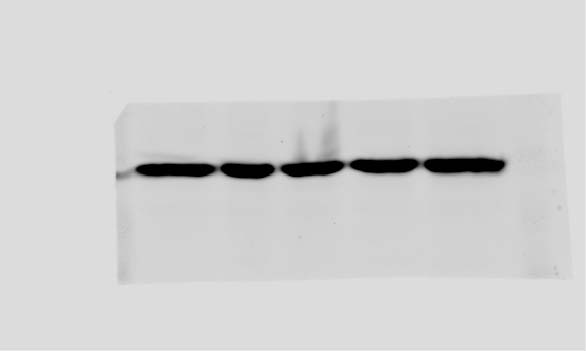


**C**





Control AdNC AdNR4A2

**D**


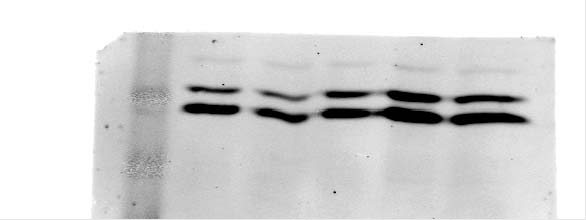


**E**


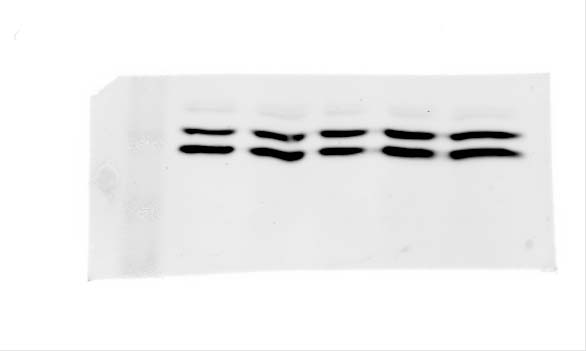


**F**





Control AdNC AdNR4A2

**Figure 4.** AdNR4A2 treatment increases the phosphorylation of ERK1/2 and P38 in hepatic stellate cells. Western blots were performed in HSC-T6 cells treated with AdNR4A2 and AdNC respectively at an MOI of 40 for 72 hours. Western blot analysis image for p-P38(A), P38 (B), gapdh (C), p-ERK(D), ERK (E) and GAPDH(F).
